# Multiple Mechanisms of Action of Sulfodyne^®^, a Natural Antioxidant, against Pathogenic Effects of SARS-CoV-2 Infection

**DOI:** 10.3390/antiox13091083

**Published:** 2024-09-04

**Authors:** Paul-Henri Romeo, Laurine Conquet, Sébastien Messiaen, Quentin Pascal, Stéphanie G. Moreno, Anne Bravard, Jacqueline Bernardino-Sgherri, Nathalie Dereuddre-Bosquet, Xavier Montagutelli, Roger Le Grand, Vanessa Petit, Federica Ferri

**Affiliations:** 1 Laboratory on Repair and Transcription in Hematopoietic Stem Cells (LRTS/IRCM), Université Paris Cité, Inserm, CEA, 92265 Fontenay-aux-Roses, France; paul-henri.romeo@cea.fr (P.-H.R.); stephanie.moreno@cea.fr (S.G.M.); anne.bravard@cea.fr (A.B.); jacqueline.bernardino-sgherri@cea.fr (J.B.-S.); 2 Laboratory on Repair and Transcription in Hematopoietic Stem Cells (LRTS/IRCM), Université Paris-Saclay, Inserm, CEA, 92265 Fontenay-aux-Roses, France; 3 Mouse Genetics Laboratory, Université Paris Cité, Institut Pasteur, 75724 Paris, France; laurine.conquet@pasteur.fr (L.C.); xavier.montagutelli@pasteur.fr (X.M.); 4 Laboratory on Development of the Gonads (LDG/IRCM), Université Paris Cité, Inserm, CEA, 92265 Fontenay-aux-Roses, France; sebastien.messiaen@cea.fr; 5 Laboratory on Development of the Gonads (LDG/IRCM), Université Paris-Saclay, Inserm, CEA, 92265 Fontenay-aux-Roses, France; 6 Center for Immunology of Viral, Auto-Immune, Hematological and Bacterial Diseases (IMVA-HB/IDMIT), Université Paris-Saclay, Inserm, CEA, 92265 Fontenay-aux-Roses, France; quentin.pascal@cea.fr (Q.P.); nathalie.bosquet@cea.fr (N.D.-B.); roger.le-grand@cea.fr (R.L.G.)

**Keywords:** Nrf2, SARS-CoV-2, COVID-19, inflammation, interferon-beta

## Abstract

Few therapeutic options are available to treat COVID-19. The KEAP1/NRF2 pathway, the major redox-responsive pathway, has emerged as a potential therapeutic target for COVID-19 as it regulates redox homeostasis and inflammation that are altered during SARS-CoV-2 infection. Here, we characterized the effects of NRF2-agonist Sulfodyne^®^, a stabilized natural Sulforaphane, in cellular and animal models of SARS-CoV-2 infection. In pulmonary or colonic epithelial cell lines, Sulfodyne^®^ elicited a more efficient inhibition of SARS-CoV-2 replication than NRF2-agonists DMF and CDDO. This antiviral activity was not dependent on NRF2 but was associated with the regulation of several metabolic pathways, including the inhibition of ER stress and mTOR signaling, which are activated during SARS-CoV-2 infection. Sulfodyne^®^ also decreased SARS-CoV-2 mediated inflammatory responses by inhibiting the delayed induction of IFNB1 and type I IFN-stimulated genes in infected epithelial cell lines and by reducing the activation of human by-stander monocytes recruited after SARS-CoV-2 infection. In K18-hACE2 mice infected with SARS-CoV-2, Sulfodyne^®^ treatment reduced both early lung viral load and disease severity by fine-tuning IFN-beta levels. Altogether, these results provide evidence for multiple mechanisms that underlie the antiviral and anti-inflammatory activities of Sulfodyne^®^ and pinpoint Sulfodyne^®^ as a potent therapeutic agent against pathogenic effects of SARS-CoV-2 infection.

## 1. Introduction

The most aggressive form of Acute Respiratory Distress Syndrome (ARDS) caused by SARS-CoV-2 is characterized by a cytokine storm and leukopenia [1]. Whereas vaccination is the most effective prophylactic treatment against SARS-CoV-2 infection, other therapeutic approaches are still needed for patients who develop pathological effects after SARS-CoV-2 infection. Monoclonal antibodies, convalescent plasma, immunomodulators, and antiviral drugs [2] are administered to patients with moderate/severe COVID-19, but most of them cannot be used at large scale due to issues with expensive costs, route of administration, and/or concerns about side effects. Thus, oral therapeutics with easily accessible drugs that decrease the symptomatology associated with SARS-CoV-2 infection are urgently required. 

The KEAP1/NRF2 pathway is the major redox-responsive pathway. Its activation protects cells against oxidative stress and damage and represses pro-inflammatory cytokine genes [3,4,5]. NRF2 transcriptionally regulates many genes coding for antioxidant proteins and proteins that regulate glutathione metabolism [6]. In addition, NRF2 has antiviral properties that can be separated from its anti-inflammatory/cytoprotective properties [7]. SARS-CoV-2 infection and replication are associated with oxidative stress that predicts disease severity in infected patients [8,9,10], and the KEAP1/NRF2 pathway is down-regulated by SARS-CoV-2. In accordance with this, analyses of lung biopsies from COVID-19 patients show that genes regulated by NRF2 have decreased mRNA levels [11] and expression of NRF2 protein is decreased in children infected with SARS-CoV-2 [12]. Furthermore, the NSP14 protein of SARS-CoV-2 impairs NRF2/HMOX1 activation [13]; the SARS-CoV-2 ORF3a positively regulates ferroptosis by degradation of NRF2 [14] and SARS-CoV-2 directly inhibits NRF2-mediated antioxidant response [15]. Interestingly, SARS-CoV-2 replication does not seem to be dependent on the KEAP1/NRF2 pathway, but the pathogenic consequences of SARS-CoV-2 infection depend on the KEAP1/NRF2 pathway [15]. These results indicate that activating the KEAP1/NRF2 pathway may be a therapeutic approach during SARS-CoV-2 infection and thus could at least complement an antiviral therapy. 

There are numerous NRF2-activating drugs used in clinical trials that are good candidates for treating the pathogenic consequences of SARS-CoV-2 infection. PB125^®^, a phytochemical NRF2-activating ingredient, inhibits SARS-CoV-2 entry by downregulating ACE2 and TMPRSS2, two SARS-CoV-2 entry factors, and downregulates several inflammatory cytokines that are activated in severe COVID-19 [16]. Dimethyl fumarate (DMF) and 4-octyl-itaconate (4-OI) inhibit viral replication in epithelial cells and decrease the expression of associated inflammatory genes when used in prophylactic treatment in vitro [11]. Bardoxolone methyl (CDDO-Me) possesses ananti-inflammatory properties against viruses like DENV or Zika virus [17]. Finally, Sulforaphane (SFN) inhibits SARS-CoV-2 replication in cell lines and displays anti-inflammatory action during SARS-CoV-2 infection [18]. SFN is a naturally occurring isothiocyanate that suppresses oxidative stress by enhancing NRF2 signaling through direct targeting of specific cysteine sensors within KEAP1, thereby releasing NRF2 [19,20,21]. SFN anti-inflammatory effect is not limited to the canonical activation of NRF2 but is also due to direct inhibition of nuclear factor kappa B (NF-κB), the catalytic activities of NF-κB kinase subunit β (IKKβ) or IKKα and STAT3 [22,23,24,25,26,27,28]. 

Even though NRF2 is its primary mediator, the antiviral activity against SARS-CoV-2 of SFN in epithelial cell lines is conserved in NRF2-knockdown cells [18], and its action may be related to its reversible inhibition of 3-chymotrypsin-like protease of SARS-CoV-2 [29], its inhibition of the NF-kB pathway [30] and/or its inhibitory effect on NRLP3 inflammasome activation [31]. These results show numerous and complex molecular mechanisms of action of SFN and that NRF2 contribution in the SFN action during SARS-CoV-2 infection remains elusive. Thus, characterizing the metabolic pathways regulated by SFN is crucial to developing targeted therapies against SARS-CoV-2 infection. 

To address these points, we investigated the effects of the natural compound Sulfodyne^®^, a stabilized SFN preparation that provides optimal bioavailability [32], on SARS-CoV-2 infection in vitro and in vivo. We reported that Sulfodyne^®^ has NRF2-independent antiviral and anti-inflammatory activities, identified metabolic pathways that underlie these NRF2-independent activities, and showed that, in vivo, Sulfodyne finely tunes IFN-beta expression and decreases the early and late pathological consequences of SARS-CoV-2 infection. Overall, this study provides mechanistic explanations of the action of Sulfodyne^®^ during SARS-CoV-2 infection and suggests that Sulfodyne^®^ is a potential therapeutic agent of COVID-19 pathogenesis.

## 2. Results

### 2.1. Increased Susceptibility of Nrf2 KO Mice to SARS-CoV-2 Infection

Respiratory viral infections, including SARS-CoV-2 infection, result in inflammation and oxidative injury. To characterize the role of NRF2, the master regulator of the antioxidant response, during SARS-CoV-2 infection in vivo, we used mice genetically deficient in NRF2 (*Nrf2* KO). *Nrf2* KO and C57BL/6 wild-type (WT) mice were infected with the Beta SARS-CoV-2 variant (Figure S1A), and clinical development of the disease was monitored for 14 days post-infection (dpi). Quantification of viral load in the lung showed significantly higher levels in *Nrf2* KO than in WT mice at 6 dpi (Figure S1B), and *Nrf2* KO mice displayed a significantly increased loss of body weight and a longer time to return to their pre-infection body weight (Figure S1C). Furthermore, whereas WT mice did not show any clinical symptoms, *Nrf2* KO mice exhibited signs of pathological infection (measured by ruffled fur, hunched posture, reduced locomotion, and difficult breathing) with maximal score at 3 dpi, indicating an increased susceptibility of *Nrf2* KO mice to SARS-CoV-2-induced disease (Figure S1D). Finally, histopathological analysis of lungs at 6 dpi showed an increase in pulmonary lesions and a more severe bronchial inflammation in *Nrf2* KO mice compared to WT (Figure S1E). 

Altogether, these results indicate an increased pathological response of *Nrf2* KO mice to SARS-CoV-2 infection and suggest that NRF2 contributes to the antiviral response against SARS-CoV-2 and/or to the pathological effects associated with SARS-CoV-2 infection. 

### 2.2. Sulfodyne^®^ Exhibits Strong Antiviral Activity in Epithelial Cell Lines

Results from *Nrf2* KO mice prompted us to investigate the effect of pharmacological activation of NRF2 on SARS-CoV-2 infection. Using Calu-3 cells, a lung human epithelial cell line highly permissive for SARS-CoV-2 infection, we studied the effect of three NRF2 agonists: the clinically approved Dimethyl Fumarate (DMF), the synthetic triterpenoid CDDO-Imidazolide (CDDO) and the natural compound Sulfodyne^®^, a stabilized Sulforaphane (SFN) preparation that provides optimal bioavailability. First, we showed that Sulfodyne^®^ treatment induced NRF2 nuclear translocation in Calu-3 cells when used at a concentration equivalent to 14 uM of SFN (Figure S2A) without affecting cell viability (Figure S2B).

**Figure 1:**
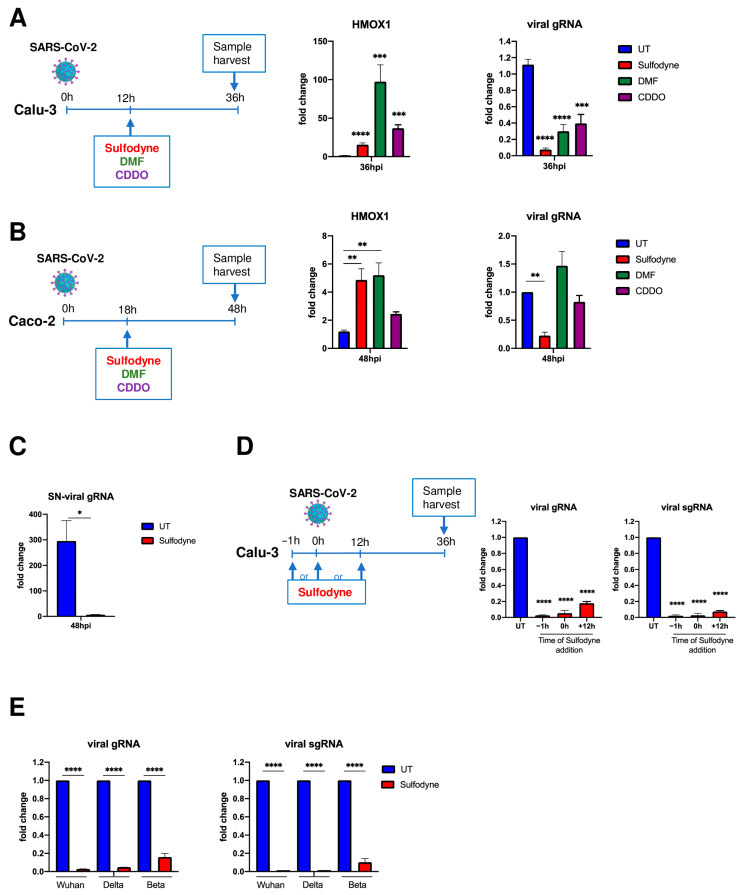
Sulfodyne^®^ efficiently inhibits SARS-CoV-2 replication in epithelial cells. (**A**) Calu-3 and (**B**) Caco2 cells were infected with SARS-CoV-2 (Wuhan) at MOI of 0.5 and were untreated (UT) or treated with indicated NRF2 activators at 12 h (Calu-3) or 18 h (Caco-2) post-infection (hpi). Viral genomic RNA (viral gRNA, primers targeting the RdRp gene in ORF1) and cellular *HMOX1* (a known NRF2 target gene) mRNA were quantified 36 hpi (Calu-3) or 48 hpi (Caco2) by RT-qPCR. Data are expressed as fold change over the UT. Mean ± sem. Calu-3: n = 11 for UT, Sulfodyne^®^ and DMF; *n* = 2 for CDDO. Caco2: *n* = 6 for UT, Sulfodyne^®^ and DMF; *n* = 2 for CDDO. One-way ANOVA with Dunnett’s multiple comparisons test vs. UT. (**C**) Expression of viral genomic RNA at 48 hpi in supernatant (SN) from Calu-3 cells infected with SARS-CoV-2 at MOI of 0.5 and untreated (UT) or treated with Sulfodyne^®^ at 12 hpi. Data are expressed as fold change over the inoculum. Mean ± sem. *n* = 3. Unpaired *t*-test. (**D**) Time-of-Sulfodyne^®^ treatment-assay. Calu-3 cells were infected with SARS-CoV-2 at MOI of 0.5 and treated with Sulfodyne^®^ at indicated times before (−1 h), at the time (0 h), or after infection (12 h). Viral genomic and sub-genomic (viral sgRNA, primers spanning the Leader sequence and the E-gene) RNA was quantified at 36 hpi and expressed as fold change over the UT. Mean ± sem; *n* = 2. One-way ANOVA with Dunnett‘s multiple comparisons test vs. UT at 36 hpi. (**E**) Calu-3 were infected with Wuhan and a variant of concern Delta and Beta SARS-CoV-2 at MOI of 0.5, treated with Sulfodyne^®^ at 12 hpi and collected at 36 hpi for viral genomic and sub-genomic RNA quantification. Data are shown relative to the expression of UT at 36 hpi. Mean ± sem; *n* = 2. One-way ANOVA with Sidak’s multiple comparisons test vs. the corresponding UT at 36 hpi. * *p* ≤ 0.05; ** *p* ≤ 0.01; *** *p* ≤ 0.001; **** *p* ≤ 0.0001.

To evaluate the antiviral activity of these NRF2 agonists, Calu-3 cells were infected with the initial pandemic SARS-CoV-2 Wuhan strain (hereafter referred to as SARS-CoV-2) at MOI 0.5 for 12 h before drug addition (Figure 1A, left panel). All drugs activated NRF2, as illustrated 36 h post-infection (hpi), by the increased mRNA level of HMOX1, a known NRF2 target gene (Figure 1A, middle panel). Quantification of intracellular genomic viral RNA expression showed that Sulfodyne^®^ was a potent suppressor of SARS-CoV-2 infection of Calu-3 cells, whereas DMF and CDDO displayed lower antiviral activity (Figure 1A, right panel). In addition, Sulfodyne^®^ but neither DMF nor CDDO decreased SARS-CoV-2 infection in the human colonic epithelial Caco2 cells, whereas all drugs activated the KEAP1/NRF2 pathway (Figure 1B). We thus focused our studies on the effect of Sulfodyne^®^ on SARS-CoV-2 infection.

The levels of genomic viral RNA load in culture supernatants of Calu-3 cells showed that the decreased intracellular viral RNA after Sulfodyne^®^ treatment was associated with a decreased SARS-CoV-2 spread (Figure 1C). If added prior to, at the time of, or after SARS-CoV-2 infection, Sulfodyne^®^ activated the KEAP1/NRF2 pathway (Figure S2C) and was highly effective (>95% of inhibition) in reducing genomic and sub-genomic SARS-CoV-2 expression (Figure 1D). We finally studied the antiviral activity of Sulfodyne^®^ against the Delta and Beta SARS-CoV-2 variants. Sulfodyne^®^ treatment decreased viral genomic and sub-genomic expression in Calu-3 cells infected with Delta and Beta strains with comparable efficacy to that reported to reference Wuhan strain (Figure 1E), indicating that Sulfodyne^®^ may inhibit replication of SARS-CoV-2 variants.

Altogether, these results showed a strong antiviral activity of Sulfodyne^®^ against SARS-CoV-2 infections in pulmonary or colonic epithelial cell lines.

### 2.3. Sulfodyne^®^ Inhibits SARS-CoV-2 Replication

Whereas NRF2 deficiency increases ACE2 expression [33], activation of NRF2 decreases ACE2 expression [16,33] and increases thioredoxin reductase (TRXR), which could reduce the interaction between SARS-CoV-2 Spike protein and ACE2 and thus decrease SARS-CoV-2 entry [34].

**Figure 2:**
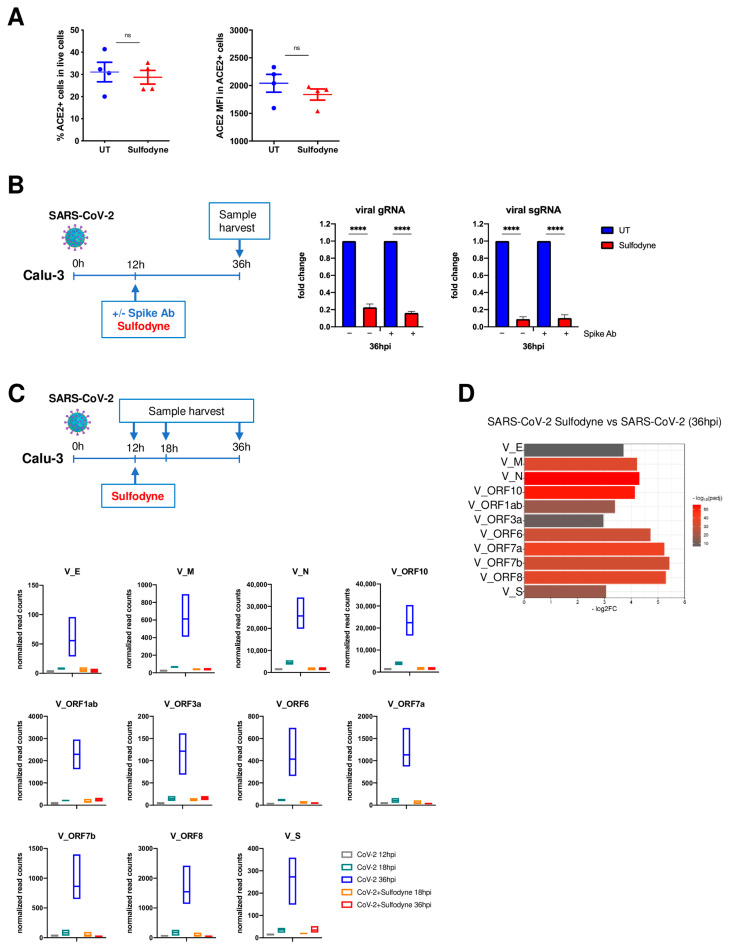
Sulfodyne^®^ blocks SARS-CoV-2 replication at SARS-CoV-2 post-entry stage. (**A**) Calu-3 cells were untreated (UT) or treated with Sulfodyne^®^ for 48 h, and ACE2 expression at the membrane of live Calu-3 cells (**Left**) and ACE2 mean fluorescence intensity (MFI) in ACE2 positive cells (**right**) were studied by flow cytometry. Mean ± sem; *n* = 4. Two-tailed Mann-Whitney U test, ns: not significant. (**B**) Calu-3 cells were infected with SARS-CoV-2 for 12 h. SARS-CoV-2 was then washed, Sulfodyne^®^ was added in the presence of the anti-Spike neutralizing antibody (Spike Ab), and genomic and sub-genomic viral RNA were quantified at 36 hpi. Data are presented as a fold decrease relative to the corresponding UT sample. Mean ± sem; *n* = 3. One-way ANOVA with Sidak’s multiple comparisons test vs. corresponding UT at 36 hpi. **** *p* ≤ 0.0001. (**C**) (**Upper** panel) Calu-3 cells were infected with SARS-CoV-2, treated with Sulfodyne^®^ at 12 hpi, and collected at the indicated time for RNAseq analysis. (**Lower** panel) Normalized read counts in Calu-3 cells of indicated RNA-seq samples for each individual SARS-CoV-2 transcript. *n* = 4. (**D**) Fold decrease of viral transcripts at 36 hpi in Calu-3 cells infected with SARS-CoV-2 and treated with Sulfodyne^®^ compared to SARS-CoV-2 infected cells. Colors represent *p*-adj from DEseq2 analysis.

To investigate if the antiviral activity of Sulfodyne^®^ was due to an impaired SARS-CoV-2 entry process to host cells, we studied the effect of Sulfodyne^®^ on cell-surface expression of ACE2, the main SARS-CoV-2 receptor in Calu-3 cells. No significant change in the percentage of ACE2-expressing Calu-3 cells nor in the ACE2 expression level at the surface of ACE2-positive cells was observed after Sulfodyne^®^ treatment (Figure 2A). To further characterize the Sulfodyne^®^ mode of action, Calu-3 cells were infected with SARS-CoV-2 for 12 h before Sulfodyne^®^ treatment, in the presence or absence of an anti-SARS-CoV-2 Spike antibody that blocks viral entry (Figure S3) and thus prevents reinfection. Quantification of intracellular viral RNA at 36 hpi showed a similar decrease in both genomic and sub-genomic RNA in the presence or absence of anti-Spike antibody in Sulfodyne^®^-treated compared to untreated cells (Figure 2B). These results indicate that Sulfodyne^®^ did not modify SARS-CoV-2 entry in Calu-3 cells. 

To characterize the kinetics of inhibition of SARS-CoV-2 replication by Sulfodyne^®^, a RNA-seq experiment was performed over a time course of 12 h, 18 h, and 36 h in Calu-3 cells infected with SARS-CoV-2 and treated with Sulfodyne^®^ at 12 hpi (Figure 2C, upper panel). The number of reads mapping to SARS-CoV-2 genes increased over time, indicating an active replication (Figure 2C, lower panels). In Sulfodyne^®^-treated Calu-3 cells, no significant increase of viral transcripts was observed between 18 and 36 hpi (Figure 2C, lower panels), indicating that Sulfodyne^®^ treatment at 12 hpi efficiently impaired the increased transcription of all viral transcripts, resulting in a 3–5 log2 fold decrease of the viral transcripts at 36 hpi compared to SARS-CoV-2 infected Calu-3 cells (Figure 2D). These results indicate that Sulfodyne^®^ greatly inhibited SARS-CoV-2 replication in Calu-3 cells.

### 2.4. Sulfodyne^®^ Regulates Host Metabolic Pathways during SARS-CoV-2 Infection

Few transcriptional changes of endogenous genes were observed in Calu-3 cells 12 h and 18 h after SARS-CoV-2 infection (respectively, 15 and 36 differentially expressed genes, *p*-adj < 0.05), whereas 500 differentially expressed genes (DEG, *p*-adj < 0.05) were identified at 36 hpi. Sulfodyne^®^ reversed the expression of genes up-regulated by SARS-CoV-2 infection at 36 dpi (Figure S4A), which are mainly genes associated with interferon (IFN) and antiviral defense pathways (Figure S4B, upper panel). Genes down-regulated by SARS-CoV-2 infection were mainly associated with the mitochondrial electron transport chain/oxidative phosphorylation (Figure S4B, lower panel). Sulfodyne^®^ treatment reversed expression of about 35% of genes that were down-regulated by SARS-CoV-2 infection at 36 dpi (Figure S4A), suggesting a partial effect of Sulfodyne^®^ on oxidative phosphorylation. 

**Figure 3:**
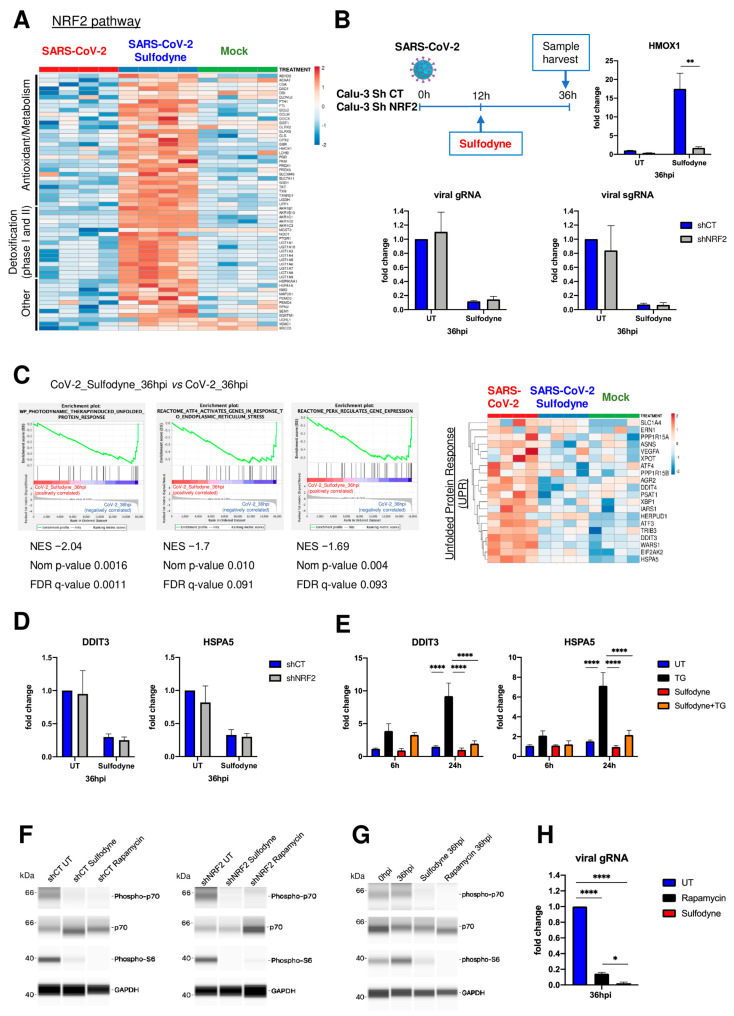
Characterization of the pathways regulated by Sulfodyne^®^ in Calu-3 cells infected with SARS-CoV-2. (**A**) Heatmap of normalized expression levels of DEG (SARS-CoV-2 +Sulfodyne^®^ vs. SARS-CoV-2 at 36 hpi) related to the NRF2 pathway in Calu-3 cells. Normalized counts are log-transformed, centered, and scaled by row. The columns display the data for each of the 4 replicates. (**B**) shCT and shNRF2 Calu-3 cells were infected with SARS-CoV-2 and treated with Sulfodyne^®^ at 12 hpi. *HMOX1* and viral RNA expression were quantified at 36 hpi and shown relative to the UT shCT. Mean ± sem; *n* = 3. Two-way ANOVA with Sidak’s multiple comparisons test vs. the corresponding shCT. (**C**) (**Left**) Gene set enrichment analysis (GSEA) showing ER stress/UPR-related pathways that are negatively correlated in SARS-CoV-2 + Sulfodyne^®^ (red) vs. SARS-CoV-2 (blue) at 36 hpi. NES, normalized enrichment score. Nom *p*-value= nominal *p*-value. FDR, False Discovery Rate. (**Right**) Heatmap of normalized expression levels of DEG related to the UPR. (**D**) *DDIT3* and *HSPA5* mRNA levels at 36 hpi in shCT and shNRF2 Calu-3 infected and treated with Sulfodyne^®^ at 12 hpi. Data are expressed as fold change over UT shCT. Mean ± sem; *n* = 3. (**E**) *DDIT3* and *HSPA5* mRNA levels in Calu-3 were treated with thapsigargin (TG), Sulfodyne^®^, and a combination of Sulfodyne^®^ + TG for 6 h and 24 h. Data are expressed as fold change over UT at 6 h. Mean ± sem; *n* = 4. Two-way ANOVA with Tukey’s multiple comparisons test. (**F**) WES Simple assay for mTOR substrate proteins phospho-p70 S6 kinase (Thr389; phospho-p70), p70 S6 kinase (p70), phospho-S6 ribosomal protein (Ser235/236; phospho-S6) and GAPDH in total extracts from shCT and shNRF2 Calu-3 cells treated with Sulfodyne^®^ or Rapamycin for 6 h. (**G**) WES Simple assay for mTOR substrate proteins and GAPDH in total extracts from Calu-3 cells noninfected (0 hpi), infected with SARS-CoV-2 (36 hpi) or infected for 12 h and then treated with Sulfodyne^®^ (Sulfodyne^®^ 36 hpi) or Rapamycin (Rapamycin 36 hpi). (**H**) SARS-CoV-2 genomic RNA levels in Calu-3 cells infected and treated as in (**G**), relative to UT at 36 hpi. Mean ± sem; *n* = 2. One-way ANOVA with Tukey’s multiple comparisons test. * *p* ≤ 0.05; ** *p* ≤ 0.01; **** *p* ≤ 0.0001.

The expression of genes regulated by the NRF2 pathway was not significantly modified during the first 36 h of Calu-3 cell infection by SARS-CoV-2 (Figure 3A). By contrast, Sulfodyne^®^ treatment during infection led to a significant induction of ARE-containing cytoprotective genes, coding for key components in antioxidant systems (Glutathione- and thioredoxin-based systems; heme and iron metabolism), drug detoxification (NQO1; UDP-glucuronosyltransferase UGT), chaperones involved in protein folding (HSP90AA1, HSPA1A) and components of proteasome (PSMD3, PSMD4) (Figure 3A).

To characterize the role of NRF2 in the antiviral action of Sulfodyne^®^, NRF2 expression was down-regulated in Calu-3 cells using shRNA (Figure S4C). As expected, NRF2 knock-down impaired the increased HMOX1 mRNA levels observed after Sulfodyne^®^ treatment (Figure 3B, upper right panel). In accordance with the transcriptomic data, no significant effect on SARS-CoV-2 replication in Calu-3 cells was observed following NRF2 knock-down (Figure 3B, lower panels) but, surprisingly, NRF2 knock-down did not modify the decrease in genomic and sub-genomic RNA expression observed after Sulfodyne^®^ treatment of SARS-CoV-2 infected Calu-3 cells (Figure 3B, lower panels). Similar results were obtained when NRF2 was knocked down in Caco2 cells (Figure S4D,E). These results indicate that NRF2 activation and, therefore, its antioxidant function by Sulfodyne^®^ treatment is not required for its antiviral action and prompted us to characterize pathways regulated by Sulfodyne^®^ and involved in its antiviral action.

Transcriptomic analyses at 36 hpi revealed a significant enrichment of pathways related to endoplasmic reticulum (ER) stress and the unfolded protein response (UPR) among genes that are up-regulated during SARS-CoV-2 infection and down-regulated after Sulfodyne^®^ treatment (Figure 3C). mRNA levels of UPR genes, including the UPR initiation marker HSPA5 (also known as BIP/GPR78) and critical transcription factors such as XBP1, ATF4, and DDIT3, were increased after SARS-CoV-2 infection, and these increases were reverted after Sulfodyne^®^ treatment at 12 hpi (Figure 3C, right panel). This Sulfodyne^®^-dependent inhibition of HSPA5 and DDIT3 increase during SARS-CoV-2 infection was also observed in NRF2 knock-down Calu-3 cells (Figure 3D), indicating a NRF2-independent action of Sulfodyne^®^ on the ER stress. As the decreased expression of UPR genes observed after Sulfodyne^®^ treatment might be a consequence of decreased viral replication, ER stress was chemically activated in Calu-3 cells by the ER stress inducer thapsigargin (TG), and the effect of Sulfodyne^®^ was studied. TG treatment activated the ER stress as shown by increased DDIT3 and HSPA5 mRNA levels, and Sulfodyne^®^ treatment impaired the TG-mediated increased levels of these mRNAs (Figure 3E), indicating that Sulfodyne^®^ can directly act on ER stress.

It has been suggested that SARS-CoV-2 activates the mTOR (mammalian Target Of Rapamycin) pathway during infection [35]. We therefore studied if Sulfodyne^®^ could act on the mTOR pathway to block SARS-CoV-2 replication. As a hallmark of mTOR activation, we investigated the phosphorylation of p70S6K (Thr389) and pS6 (Ser235/236), two ribosomal proteins essential for protein synthesis. Sulfodyne^®^ treatment decreased the phosphorylation of these proteins in an NRF2-independent manner, and this decrease was identical to that obtained after the action of Rapamycin, the main inhibitor of mTOR (Figure 3F). Sulfodyne^®^ treatment of Calu-3 or Caco2 cells after SARS-CoV-2 infection led to a decreased phosphorylation of p70S6k and pS6 similar to the one observed after treatment with Rapamycin (Figure 3G and Figure S4F), indicating that Sulfodyne^®^ inhibits the mTOR pathway during SARS-CoV-2 infection. Rapamycin treatment of Calu-3 cells decreased SARS-CoV-2 replication but less than Sulfodyne^®^ treatment (Figure 3H).

Altogether, these results indicate that, although the antiviral action of Sulfodyne^®^ in Calu-3 or Caco2 cells is not dependent on NRF2 expression, Sulfodyne^®^ treatment can repress SARS-CoV-2 replication by its action on several protective cellular metabolic pathways activated during SARS-CoV-2 infection, such as ER stress and mTOR signaling, and may restore redox homeostasis through activation of NRF2 target genes. 

### 2.5. Anti-Inflammatory Action of Sulfodyne^®^ Is Independent of SARS-CoV-2 Replication

**Figure 4:**
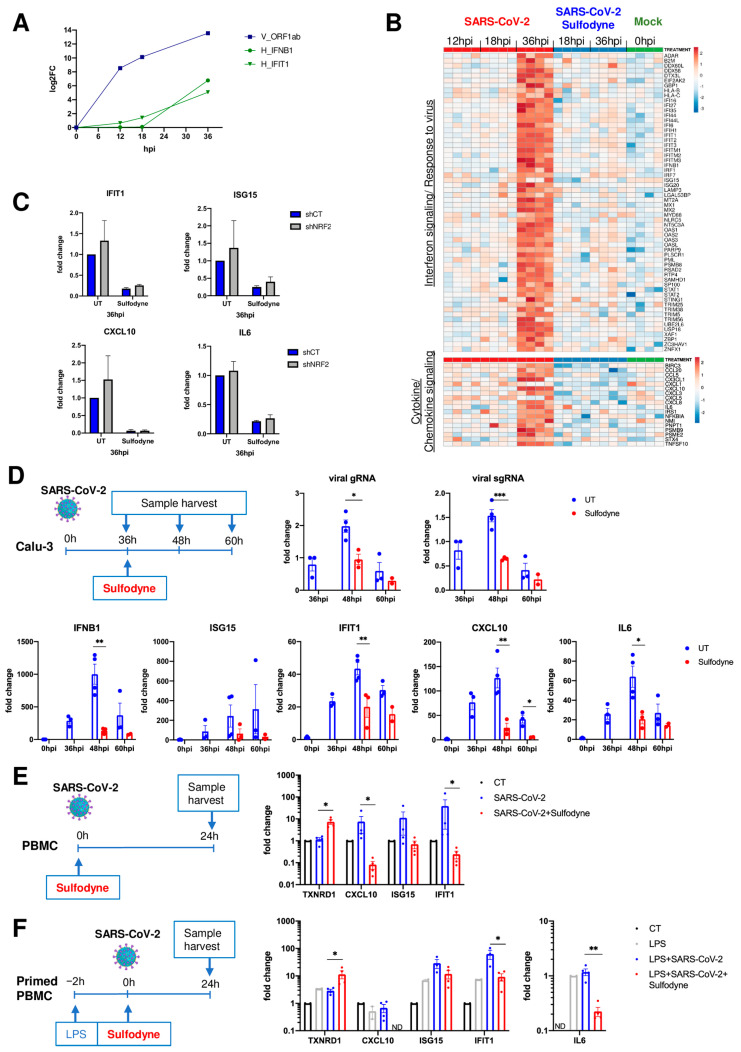
Anti-inflammatory actions of Sulfodyne^®^. (**A**) Kinetics of viral replication (ORF1ab SARS-CoV-2 gene) and of the mRNA levels of the cellular genes *IFNB1* and *IFIT1* (IFN-stimulated gene) in Calu-3 cells infected with SARS-CoV-2. Data are the mean of 4 replicates from the RNA-seq experiment and presented as Log2FC over the mock Calu-3 cells. (**B**) Heatmap of normalized expression levels of DEG (SARS-CoV-2 + Sulfodyne^®^ vs. SARS-CoV-2 at 36 hpi) related to Interferon signaling and Response to the virus (upper panel) and Cytokine and Chemokine signaling pathways (lower panel) for all RNA-seq samples in Calu-3 cells. (**C**) mRNA levels of the indicated genes in shCT and shNRF2 Calu-3 cells infected with SARS-CoV-2 and treated with Sulfodyne^®^ at 12 hpi. Data are presented relative to UT shCT at 36 hpi. Mean ± sem, *n* = 3. (**D**) Kinetics of viral RNA expression (upper panel) and of the mRNA levels of the indicated interferon-stimulated and inflammatory genes (lower panel) in Calu-3 cells infected with SARS-CoV-2 and treated with Sulfodyne^®^ at 36 hpi. Data are presented relative to the UT cells at 36 hpi for viral RNA and relative to uninfected cells (0 hpi) for host genes. Mean ± sem; *n* = 3. One-way ANOVA with Sidak’s multiple comparisons test UT vs. Sulfodyne^®^ at each time. (**E**) Human PBMC from healthy donors were mock-infected (CT), infected with SARS-CoV-2 in the presence or absence of Sulfodyne^®^ (25 μg/mL) for 24 h and analyzed for mRNA levels of the indicated genes. Data are expressed as fold change over CT PBMC. Mean ± sem, *n* = 2–4. Two-tailed Mann-Whitney U test (SARS-CoV-2 + Sulfodyne^®^ vs. SARS-CoV-2). (**F**) Human PBMC were primed with LPS before being infected and treated as in (**E**) and analyzed for mRNA levels of indicated genes. Data are expressed as fold change over CT PBMC. Mean ± sem, *n* = 2–4. Two-tailed Mann-Whitney U test (LPS + SARS-CoV-2+ Sulfodyne^®^ vs. LPS + SARS-CoV-2). ND= not detected. * *p* ≤ 0.05; ** *p* ≤ 0.01; *** *p* ≤ 0.001.

In accordance with previous results [36,37,38], we found that Calu-3 cells activate delayed IFN and antiviral defense responses relative to SARS-CoV-2 replication (Figure 4A,B, upper panel), which might also contribute to pathogenic inflammatory response. Sulfodyne^®^ treatment 12 h post-infection dampened type I IFN responses (Figure 4B, upper panel) and mRNA levels of several inflammatory genes (Figure 4B, lower panel), including genes encoding for chemo-attractants for monocytes and neutrophils. As for its action on viral infection, the anti-inflammatory activity of Sulfodyne^®^ was independent of NRF2 expression (Figure 4C).

As SARS-CoV-2 replication induced an inflammatory response in Calu-3 cells, the observed decreased mRNA levels of inflammatory genes after Sulfodyne^®^ treatment might be a consequence of decreased viral replication. To test this hypothesis, Calu-3 cells were treated with Sulfodyne^®^ at 36 hpi, a time at which Calu-3 inflammatory response is high (Figure 4B) and active viral replication is low (Figure 4A,D, upper panels). Kinetics analysis showed that Sulfodyne^®^ decreased mRNA levels of IFNB1, IFN-stimulated genes (ISG15, IFIT1), CXCL10, and IL6 (Figure 4D, lower panels), suggesting that the anti-inflammatory activity of Sulfodyne^®^ is not only due to decreased SARS-CoV-2 replication. 

Peripheral blood immune cells, recruited to the lung compartment, are major contributors to human inflammatory responses after SARS-CoV-2 infection. Infection of human peripheral blood mononuclear cells (PBMC) with SARS-CoV-2 is not productive as SARS-CoV-2 cannot replicate in PBMC, but exposure of PBMC to SARS-CoV-2 can induce an innate immune response [39]. We therefore studied the effects of Sulfodyne^®^ treatment on the inflammatory response of PBMC exposed to SARS-CoV-2. PBMC from healthy donors were exposed ex vivo to SARS-CoV-2 and treated with Sulfodyne^®^ for 24 h (Figure 4E). Sulfodyne^®^ activated NRF2 and decreased mRNA levels of CXCL10 and interferon-stimulated genes IFIT1 and ISG15, which increased after SARS-CoV-2 infection (Figure 4E). Furthermore, as SARS-CoV-2 infection of immune cells in vivo can occur in a pre-existing inflammatory environment, PBMC were primed with LPS 2 h before viral infection and Sulfodyne^®^ treatment (Figure 4F, left panel). Under this condition, Sulfodyne^®^ activated NRF2 (Figure 4F, middle panel). Exposure of PBMC to LPS alone has no effect on CXCL10 mRNA level but increased IFIT1 and ISG15 mRNA levels that were further increased following SARS-CoV-2 infection. Sulfodyne^®^ treatment only reduced the viral-dependent increase of these mRNA levels (Figure 4F, middle panel). In contrast, the IL6 mRNA level did not increase after SARS-CoV-2 infection, and Sulfodyne^®^ decreased the IL6 mRNA level (Figure 4F, right panel). 

Altogether, these results indicated that Sulfodyne^®^ treatment could decrease the inflammatory response associated with SARS-CoV-2 infection in both epithelial cells and primary immune cells independently of SARS-CoV-2 replication and NRF2 expression.

### 2.6. Immunomodulatory Impact of Sulfodyne^®^ on By-Stander Monocytes

High release of inflammatory mediators by infected lung epithelial cells may exacerbate monocyte infiltration and immune response, leading to severe COVID-19 pathological effects, and the anti-inflammatory action of Sulfodyne^®^ in lung epithelial cells could be beneficial in preventing hyperinflammatory reactions that contribute to lung damage. 

**Figure 5:**
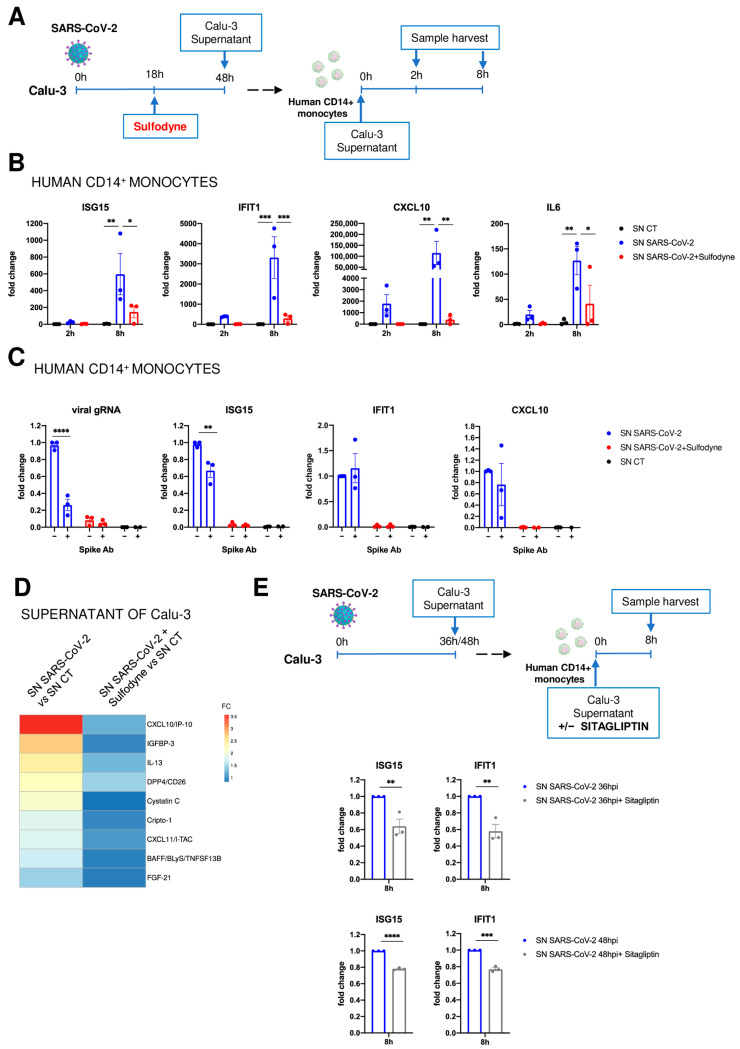
By-stander actions of Sulfodyne^®^ on human monocytes. (**A**) Schematic of experimental design. Calu-3 cells were infected with SARS-CoV-2 and treated with Sulfodyne^®^ at 18 hpi. Cellular supernatants (SN) from uninfected (SN CT), SARS-CoV-2 infected (SN SARS-CoV-2) and SARS-CoV-2 infected and Sulfodyne^®^ treated (SN SARS-CoV-2 + Sulfodyne^®^) were collected 48 hpi and used to treat human CD14^+^ monocytes during 2 h or 8 h. (**B**) mRNA levels of the indicated genes were measured in human CD14^+^ monocytes 2 h and 8 h after exposure of indicated SN. Data are presented relative to human CD14^+^ monocytes treated with SN CT for 2 h. Mean ± sem; *n* = 3. Two-way ANOVA with Tukey’s multiple comparisons test. (**C**) Indicated SN from Calu-3 was pre-incubated in the presence or not of the anti-Spike neutralizing antibody (Spike Ab) for 1 h at 37 °C before treatment of human CD14^+^ monocytes. Viral genomic RNA and mRNA levels of the indicated host genes were measured in human CD14^+^ monocytes at 8 h after exposure to indicated SN. Data are presented relative to human CD14^+^ monocytes treated with SN SARS-CoV-2 without Spike Ab. Mean ± sem; *n* = 3. Two-way ANOVA with Sidak’s multiple comparisons test vs. corresponding SN without Spike Ab. (**D**) Heatmap of soluble mediators in the cellular supernatants of SARS-CoV-2 and SARS-CoV-2 + Sulfodyne^®^ depicted in (**A**). Data are presented as fold change relative to the SN CT. (**E**) Human CD14^+^ monocytes were exposed to SN from SARS-CoV-2 infected Calu-3 cells for 36 or 48 h in the presence of Sitagliptin, a DPP4 inhibitor. mRNA levels of the indicated genes were measured 8 h later and compared to mock-treated monocytes exposed to SN from infected Calu-3 cells. Mean ± sem, n = 3 independent SN used on 2 different donors. Unpaired *t*-test. * *p* ≤ 0.05; ** *p* ≤ 0.01; *** *p* ≤ 0.001; **** *p* ≤ 0.0001.

We, therefore, assessed if SARS-CoV-2 infection of lung epithelial cells can promote an inflammatory response in human CD14^+^ monocytes and studied the effect of Sulfodyne^®^ treatment on this inflammatory response. Human CD14^+^ monocytes were purified from healthy donors and cultured for 2 h and 8 h in the presence of supernatant from uninfected, SARS-CoV-2-infected, and SARS-CoV-2-infected and Sulfodyne^®^-treated Calu-3 cells (Figure 5A). Exposure of human CD14^+^ monocytes to the supernatant of SARS-CoV-2 infected Calu-3 cells increased mRNA levels of ISG15, IFIT1, CXCL10, and IL6, and this increase was significantly reduced when supernatant from Sulfodyne^®^-treated Calu-3 cells was used (Figure 5B). The different levels of SARS-CoV-2 virions during exposure of monocytes might cause the differential effects of supernatant of SARS-CoV-2-infected Calu-3 cells on monocyte response. We thus studied the effects of neutralization of SARS-CoV-2 Spike in supernatants from SARS-CoV-2-infected Calu-3 on monocyte response. As expected, the SARS-CoV-2 Spike neutralization efficiently decreased SARS-CoV-2 entry in monocytes (Figure 5C, left panel) but only resulted in a slight reduction of ISG15 and no significant changes of IFIT1 and CXCL10 mRNA levels in by-stander monocytes (Figure 5C, right panels). These results indicated that the immunomodulatory effect of Sulfodyne^®^ in bystander monocytes was not dependent on exposure of these monocytes to SARS-CoV-2 virions. 

We thus studied the proteins present in the supernatant of SARS-CoV-2 infected Calu-3 cells and potentially involved in monocyte activation. Proteome profiling of Calu-3 infected or non-infected supernatants used to treat human monocytes showed increased protein levels of several inflammatory mediators, which were decreased in supernatants of Sulfodyne^®^-treated cells (Figure 5D). Among these inflammatory mediators, we identified known markers of severe COVID-19, such as the monocyte-recruiting chemokine CXCL10 and DPP4 [40,41]. Decreased levels of secreted CXCL10 after Sulfodyne^®^ treatment (Figure 5D) were associated with its decreased mRNA expression (Figure 4B). DPP4, a multifunctional cell-membrane protease, has a role in glucose metabolism [42], but increasing evidence suggests that its soluble form can act on innate immune responses and inflammation [43]. In supernatants, only the soluble form of DPP4 (sDPP4) could be detected, and Sulfodyne^®^ decreased levels of sDPP4 without any change in DPP4 mRNA expression (Figure S5A). To characterize the role of sDPP4 present in the supernatant of SARS-CoV-2-infected Calu-3 cells on monocytes, these were treated with Sitagliptin, a specific inhibitor of DPP4, during exposure of monocytes to supernatant from SARS-CoV-2-infected Calu-3 cells (Figure 5E, upper scheme). Sitagliptin reduced the increased levels of ISG15 and IFIT1 mRNAs in the exposed monocytes (Figure 5E, lower panels). As no DPP4 expression could be detected in monocytes (Figure S5B), these results suggested a contribution of secreted DPP4 in monocyte activation and pinpointed how Sulfodyne^®^ could decrease the inflammatory response associated with SARS-CoV-2 infection. 

### 2.7. Sulfodyne^®^ Treatment Confers Protection against SARS-CoV-2 Infection In Vivo

We investigated the in vivo effects of Sulfodyne^®^ in the Syrian golden hamster animal model of SARS-CoV-2 infection [44]. Hamsters were treated orally with Sulfodyne^®^ (600 mg/kg) 8 h after intranasal inoculation of SARS-CoV-2 and twice a day for 3 days (Figure S6A). Whereas Sulfodyne^®^ treatment did not decrease viral load in the lungs at 3 dpi (Figure S6B), it prevented loss of body weight after SARS-CoV-2 infection (Figure S6C), suggesting a protective role of Sulfodyne^®^ against the pathological effects due to SARS-CoV-2 infection.

**Figure 6:**
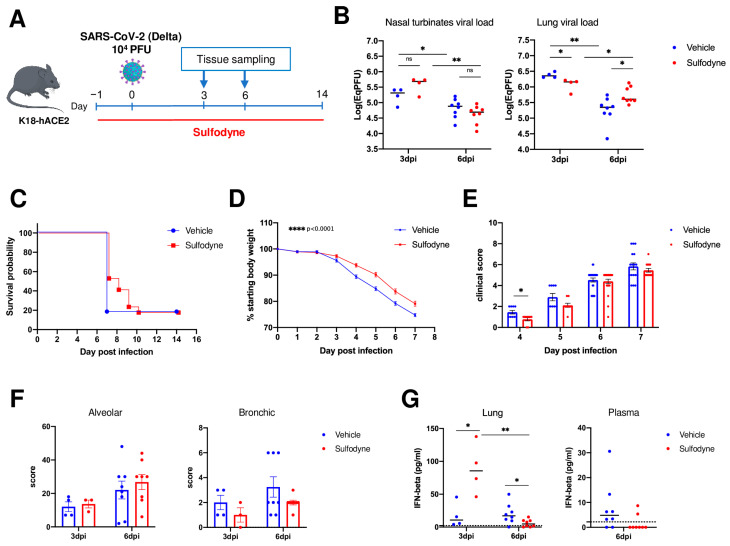
In vivo actions of Sulfodyne^®^ on SARS-CoV-2 symptomatology. (**A**) Schematic of experimental design. K18-hACE2 mice were intraperitoneally injected with Sulfodyne^®^ (600 mg/kg) or vehicle (PBS 2%EtOH) one day prior to intranasal infection with 10^4^ PFU of SARS-CoV-2 Delta variant. Sulfodyne^®^ or vehicles were then administered twice a day through the end of the study. (**B**) Viral load quantification in homogenized nasal turbinates (**left**) and lung (**right**) tissue at 3 and 6 dpi in the vehicle and Sulfodyne^®^-treated mice. Individual values for each mouse and median are presented. n = 4 at 3 dpi; n = 8 at 6 dpi. Two-tailed Mann-Whitney U test. (**C**) Kaplan–Meier plot of survival of vehicle and Sulfodyne^®^-treated K18-hACE2 mice. n = 17. (**D**) Body weight change during the course of infection presented as a percent change compared to weight measured just before inoculation with SARS-CoV-2. Mean ± sem, n = 29. Mixed-effects model comparing Sulfodyne^®^ vs. vehicle-treated mice. (**E**) The clinical score was assessed for ruffled fur, hunched posture, reduced locomotion, and difficulty breathing, and the score ranged from 0 to 2. The cumulative clinical score is indicated. Mean ± sem, n = 9 to 25. Two-tailed Mann-Whitney U test. (**F**) Alveolar (**left**) and bronchic (**right**) histopathological scores in the vehicle and Sulfodyne^®^-treated mice at 3 and 6 dpi. n = 4 (vehicle) or 3 (Sulfodyne^®^) at 3 dpi; n = 8 at 6 dpi. (**G**) IFN-beta levels in lung homogenates (**left**) and plasma (**right**) from the vehicle and Sulfodyne^®^-treated mice at 3 and 6 dpi. n = 4 at 3 dpi; n = 8 at 6 dpi. Two-tailed Mann-Whitney U test. ns: not significant, * *p* ≤ 0.05; ** *p* ≤ 0.01; **** *p* ≤ 0.0001.

We then studied the effects of Sulfodyne^®^ in transgenic mice expressing the human ACE2 under the control of Keratin18 promoter (K18-hACE2) that provides a reliable mouse model for SARS-CoV-2 infection and associated severe COVID-19 disease [45]. K18-hACE2 mice were infected intranasally with the Delta variant of SARS-CoV-2 and treated intraperitoneally (i.p.) with Sulfodyne^®^ (600 mg/kg) twice a day, starting one day before infection and for up to 14 days after infection (Figure 6A). Sulfodyne^®^ treatment did not affect SARS-CoV-2 replication in nasal turbinates (Figure 6B, left panel). However, Sulfodyne^®^ treatment resulted in a significant reduction in lung viral load at 3 dpi but not at 6 dpi, suggesting that Sulfodyne^®^ reduced early SARS-CoV-2 replication in the lung (Figure 6B, right panel). Infection of K18-hACE2 mice showed a severe course of disease with a high lethality rate at 7 dpi, and Sulfodyne^®^ treatment did not improve survival but could delay median survival by one day (Figure 6C). Nevertheless, Sulfodyne^®^ treatment significantly reduced body weight loss (Figure 6D) and decreased the early clinical symptoms (Figure 6E, day 4). Furthermore, although in Sulfodyne^®^-treated mice, the lung viral load was increased at 6 dpi compared to vehicle-treated mice (Figure 6B), no significant increase of clinical symptoms was observed at 6 and 7dpi (Figure 6E). In line with this data, Sulfodyne^®^-treated mice displayed a trend toward decreased bronchial inflammation scores at 6dpi compared to vehicle-treated animals (Figure 6F). Sulfodyne^®^ treatment significantly increased IFN-beta levels in lung homogenates at 3 dpi, suggesting that Sulfodyne^®^ might enhance the antiviral response during the early stages of SARS-CoV-2 infection (Figure 6G). In addition, at 6 dpi, Sulfodyne^®^ decreased late IFN-beta levels in both lung homogenates and plasma (Figure 6G), highlighting a protective role of Sulfodyne^®^ in reducing the inflammatory response at late stages. 

Altogether, these results pinpoint the beneficial role of Sulfodyne^®^ treatment in vivo for reducing the severity of disease associated with SARS-CoV-2 infection. 

## 3. Discussion

In this report, we identify the natural compound Sulfodyne^®^, a stabilized form of NRF2 agonist SFN, as a potent agent against SARS-CoV-2 pathogenesis and provide evidence for multiple mechanisms that underlie the Sulfodyne^®^ beneficial effects. 

As SARS-CoV-2 infection interferes with the metabolism and redox function of cellular glutathione [46], it has been suggested that the NRF2 pathway is targeted by SARS-CoV-2, leading to the reduction of NRF2-mediated antioxidant responses. Indeed, in accordance with a recent study [15], we showed that NRF2 deficiency in mice infected with SARS-CoV-2 was associated with increased lung viral load and signs of exacerbated symptomatology. However, transcriptomic analyses did not evidence modulation of expression of genes linked to the NRF2 pathway in SARS-CoV-2 infected Calu-3 cells 36 h after infection. This result extends a previous study that reported decreased NRF2 protein levels at 48 h but not 24 h after infection of Calu-3 cells [15] and is in accordance with the repression of the NRF2 pathway in COVID-19 patient lung biopsies [11], suggesting that decreased levels of NRF2 is a late event during SARS-CoV-2 infection. 

Sulfodyne^®^ treatment of epithelial cell lines elicited a more efficient inhibition of SARS-CoV-2 replication than DMF and CDDO, and this inhibition occurs at post-entry stages. Although Sulfodyne^®^ treatment resulted in strong activation of the NRF2 pathway, decreased SARS-CoV-2 replication by Sulfodyne^®^ is not dependent on NRF2 expression. Thus, we characterized the metabolic host pathways activated during SARS-CoV-2 infection and inhibited by Sulfodyne^®^. SARS-CoV-2 replication is structurally and functionally associated with the ER as SARS-CoV-2 ORF8 escapes degradation by host cells and induces ER stress by targeting ER chaperones and key UPR components [47]. Sulfodyne^®^ inhibited the ER stress and mTOR signaling pathway that is activated during SARS-CoV-2 infection for viral protein translation [48]. Thus, our study expands previous works showing new SFN targets beyond the activation of NRF2 [49,50,51]. Further studies are now needed to determine how Sulfodyne^®^ targets the ER stress and the mTOR signaling. In addition to these effects of Sulfodyne^®^ on SARS-CoV-2 replication, Sulfodyne^®^ increased expression of NRF2-target genes that activate the glutathione antioxidant system, which is important to restore cellular homeostatic processes during the inhibition of viral replication. The beneficial effects of Sulfodyne are likely due to a combination of effects of Sulfodyne^®^ on multiple host pathways essential for SARS-CoV-2 replication activation and the antioxidant system, which is important to restore cell homeostasis.

Type I IFNs are essential in the defense against SARS-CoV-2 infection, as genetic deficiencies in IFN signaling or the presence of autoantibodies neutralizing type I IFNs are strong risk factors for life-threatening COVID-19 pneumonia [52,53,54]. However, although rapid induction of type I IFNs limits virus propagation, late-onset and continuous high levels of type I IFNs disrupt adaptive humoral and cellular immune responses to SARS-CoV-2 infection [55], resulting in immunopathology in the late phase of SARS-CoV-2 infection. Clinically, high levels of type I IFNs are associated with poor outcomes [56,57,58,59,60] and disappointing results of clinical trials that used recombinant IFN therapy [61]. Sulfodyne^®^ inhibited induction of *IFNB1* and type I IFN-stimulated genes in SARS-CoV-2-infected Calu-3, in which a delayed activation of type I IFN signaling contributed to an exacerbated inflammatory response [38]. This inhibition was not restricted to epithelial cells as Sulfodyne^®^ also decreased IFN-stimulated genes in both human PBMC infected with SARS-CoV-2 and in human PBMC activated with proinflammatory stimuli prior to infection with SARS-CoV-2. In the lungs of K18-hACE2 mice infected with SARS-CoV-2, Sulfodyne^®^ increased IFN-beta levels early after infection and decreased IFN-beta levels at late stages of infection. This dual effect of Sulfodyne^®^ in IFN-beta levels might be associated with its enhanced early antiviral response and its anti-inflammatory effect at late stages of SARS-CoV-2 infection. These results indicate a therapeutic effect of Sulfodyne^®^ in the pathogenic consequences of sustained levels of type I IFNs during SARS-CoV-2 infection and suggest that high levels of interferons in patients at late stages of infection or in patients with long COVID [62] might be a biomarker for the use of Sulfodyne^®^ in clinics. 

Epithelial-immune crosstalk is involved in many aspects of the local immune response to SARS-CoV-2 infection [38,63,64]. By modeling in vitro the crosstalk between infected epithelial cells and monocytes, we showed that SARS-CoV-2 infection in Calu-3 cells creates a pro-inflammatory microenvironment that drives innate immune responses in monocytes and that this activation could be prevented by treating epithelial cells with Sulfodyne^®^. Two known markers of severe COVID-19 were identified among the soluble mediators modulated during Sulfodyne^®^ treatment, CXCL10, and DPP4. As CXCL10 increases chemoattraction and recruitment of circulating monocytes/macrophages in tissues, decreased levels of secreted CXCL10 found in supernatant from Sulfodyne^®^-treated Calu-3 indicated that Sulfodyne^®^ might prevent excessive monocyte infiltration. In addition, as we showed that DDP4 might directly activate type I IFN responses in monocytes during SARS-CoV-2 infection, Sulfodyne^®^ might also regulate type I IFN response of infiltrated monocytes. Altogether, these results provide a potential mechanism for the immunomodulatory action of Sulfodyne and might explain previous works showing the beneficial effects of SFN in reducing myeloid cell recruitment and immune cell activation in the lung of SARS-CoV-2-infected mice [18].

SFN, including the encapsulated form of Sulfodyne^®^, Prostaphane [65], is orally available and well-tolerated without significant side effects. Several clinical trials have shown its benefits in different diseases, including lung and inflammatory diseases [28]. In rats, SFN is rapidly absorbed, reaches a maximum plasma concentration of 4 h after absorption, and has a half-life of around 2 h [66]. In humans, the bioavailability of SFN is variable and depends on the concentration and type of formulations administered [67]. In different animal models, we used a dose of Sulfodyne^®^ corresponding to 30 mg/kg of SFN previously used in K18-hACE2 mice [18] without any side effects. The discrepancy between the in vitro and in vivo effects of Sulfodyne^®^ on SARS-CoV-2 replication and between the Syrian golden hamsters and K18-hACE2 mice models might be related to Sulfodyne^®^ prophylactic administration only in K18-hACE2 mice, to its mode of administration (orally in hamsters, i.p. in mice), to its bioavailability and/or the non-physiological expression of the hACE2 transgene in K18-hACE2 mice. Although our study is a promising pre-clinical study, the efficiency of Sulfodyne^®^ in patients requires further studies in humans to characterize Sulfodyne^®^ pharmacokinetic properties before any use of Sulfodyne^®^ to treat the pathogenic consequences of SARS-CoV-2 infection. 

In conclusion, by lowering the early lung viral load and by decreasing the inflammatory response associated with SARS-CoV-2 infection, Sulfodyne^®^ contributes to reducing pathological effects associated with SARS-CoV-2 infection. Thus, considering its beneficial role in conferring protection during SARS-CoV-2 infection at multiple levels, Sulfodyne^®^ appears as a promising therapeutic agent of COVID-19 symptomatology.

## 4. Materials and Methods 

### 4.1. Cell Culture, Treatments and Virus

Human airway epithelial cells Calu-3 (ATCC) were cultured in EMEM supplemented with 15% FCS. Human colorectal adenocarcinoma cells Caco-2 (ATCC) were cultured in DMEM supplemented with 10% FCS and 1% not essential amino acids. 

High-purity Sulfodyne® (https://ingoodbyolga.com/en/ingredient/sulfodyne/, accessed on 9 July 2024), a health ingredient of broccoli seeds extract titrated in 5% of natural, active and stabilized SFN, was provided by Ingood by Olga company (Noyal-sur-Vilaine, France), dissolved in PBS and used, unless otherwise indicated, at 50 μg/mL, a concentration equivalent to 14 μM of SFN. DMF (Sigma-Aldrich, St. Louis, MO, USA) was used at 150 μM, CDDO-Imidazolide (Sigma-Aldrich) at 50 nM, Thapsigargin (Sigma-Aldrich, St. Louis, MO, USA) at 10 μM, Rapamycin (Sigma-Aldrich, St. Louis, MO, USA) at 1 μM, Sitagliptin (Selleckchem) at 100 μM, LPS (Sigma-Aldrich, St. Louis, MO, USA) at 100 ng/mL. Neutral Red cytotoxicity assay (Sigma-Aldrich) was performed according to the manufacturer’s instructions.

SARS-CoV-2 Wuhan, Beta (B.1.351 strain) and Delta (B.1.617.2 strain) strains were provided by the National Reference Center for Respiratory Viruses (Institut Pasteur, Paris, France). All procedures involving infectious SARS-CoV-2 were performed in biosafety level 3 (BSL-3) facilities at IDMIT (CEA, Fontenay-aux-Roses, France).

### 4.2. RNA Extraction and RT-qPCR

Total cellular RNA was extracted with the nucleospin RNA plus XS (Macherey-Nagel, Allentown, PA, USA). Viral RNA in supernatant cell culture was isolated with the Nucleospin Dx virus (Macherey-Nagel, Allentown, PA, USA). Reverse transcription was performed with random primers and Superscript IV (Life Technologies, Carlsbad, CA, USA) and quantitative PCR with the Power SYBR green PCR master mix (Applied Biosystems, Waltham, MA, USA) for host transcripts or TaqMan™ Fast Advanced Master Mix (Applied Biosystems, Waltham, MA, USA) for viral transcripts. The relative quantification of mRNA levels was calculated using the threshold cycle (2−ΔΔCT) method with ACTB RNA as an internal control. 

The following primer pairs were used (forward and reverse): 

HMOX1: CCAGGCAGAGAATGCTGAGTTC and AAGACTGGGCTCTCCTTGTTGC 

TXNRD1: GTTACTTGGGCATCCCTGGTGA and CGCACTCCAAAGCGACATAGGA

HSPA5: CTGTCCAGGCTGGTGTGCTCT and CTTGGTAGGCACCACTGTGTTC

DDIT3: GGTATGAGGACCTGCAAGAGGT and CTTGTGACCTCTGCTGGTTCTG

IFNB1: TCATGAGTTTTCCCCTGGTG and GTTGAGAACCTCCTGGCTAATG

IFIT1: GCCTTGCTGAAGTGTGGAGGAA and ATCCAGGCGATAGGCAGAGATC

ISG15: CTCTGAGCATCCTGGTGAGGAA and AAGGTCAGCCAGAACAGGTCGT

CXCL10: CGCTGTACCTGCATCAGCATTAG and CTGGATTCAGACATCTCTTCTCACC

IL6: TCCAGAACAGATTTGAGAGTAGTG and GCATTTGTGGTTGGGTCAGG

ACTB: CACCATTGGCAATGAGCGGTTC and AGGTCTTTGCGGATGTCCACGT

IP4 (SARS-CoV-2 genomic): GGTAACTGGTATGATTTCG and CTGGTCAAGGTTAATATAGG, (probe) TCATACAAACCACGCCAGG; 

E gene/Leader (SARS-CoV-2 subgenomic): CGATCTCTTGTAGATCTGTTCTC and ATATTGCAGCAGTACGCACACA, (probe) ACACTAGCCATCCTTACTGCGCTTCG.

### 4.3. RNA-Seq

Calu-3 cells were infected with SARS-CoV-2 and treated or not with Sulfodyne^®^ at 12 hpi. Cells were harvested at 0 hpi (mock), 12 hpi, 18 hpi, and 36 hpi for RNA-seq. Four independent biological replicates were performed per experimental condition. Library construction, sequencing on Oxford Nanopore Technologies sequencer, and bioinformatics analysis were performed by Life & Soft company (Fontenay-aux-Roses, France). A total of 100 ng of RNA per sample was retrotranscribed by a strand-switching technique using Maxima H Minus Reverse Transcriptase (ThermoFisher, Waltham, MA, USA) to synthesize a double-stranded cDNA. PCR, barcode, and adapter attachment were performed according to the PCR-cDNA Barcoding kit (Oxford Nanopore Technologies, Oxford, UK). Samples were quantified using QuBit dsDNA HS kit (ThermoFisher, Waltham, MA, USA) before loading on R9.4.1 Flow cells using the GridION instrument (Minknow version: 21.11.6) (Oxford Nanopore Technologies, Oxford, UK). Sequence reads were converted into FASTQ files. Reads under 300 bp or with a quality score under 9 were discarded. The remaining reads were mapped on the human GRCh38.p13 and SARS-CoV-2 ASM985889v3 transcriptome of reference using minimap2 version 2.17 [68]. To quantify transcripts, the resulting alignments were given to Salmon version 1.6.0 [69]. Differentially expressed genes (DEG) were determined using DESeq2. Enrichment analysis was performed using Metascape or GSEA. 

### 4.4. Protein Extraction and Capillary-Based Immunoblotting (WES Simple Western)

Total protein extraction was performed with RIPA buffer (20 mM HEPES pH 7.6, 150 mM NaCl, 1% NP4, 0.25% sodium deoxycholate, 10% glycerol). For nuclear extracts, cells were resuspended in hypotonic buffer (10 mM HEPES pH 7.6, 1.5 mM MgCl_2_, 10 mM KCl, and protease inhibitor cocktail), and nuclei were extracted in 20 mM HEPES pH 7.6, 20% glycerol, 420 mM NaCl, 1.5 mM MgCl_2_, 0.2 mM EDTA, 0.5 mM DTT, and protease inhibitor cocktail.

Proteins were run on the WES Simple Western system using a 12–230 kDa separation module (Bio-Techne, Minneapolis, MN, USA). 

### 4.5. Flow Cytometry

Calu-3 cells were washed in PBS and stained for viability with Biolegend Zombie Aqua Fixable Viability kit and Fc Block (Myltenyi, Paris, France) for 10 min at room temperature. Cell surface staining was performed with ACE2 primary antibody at 2.5 µg/mL for 1 h at 4 °C, followed by Allophycocyanin-conjugated Anti-Goat IgG Secondary Antibody for 30 min at 4 °C in dark. Cells were then washed with 2% FCS in PBS and resuspended in 300 µL PBS. Cells were characterized using a Canto II flow cytometer (BD Bioscience, Franklin Lakes, NJ, USA) and analyzed using Flowjo v10.6.2 software (BD Bioscience, Franklin Lakes, NJ, USA).

### 4.6. Antibodies 

Antibodies used for capillary-based immunoblotting are Phospho-p70 S6 Kinase (Thr389; Cell Signaling 9205S), p70 S6 Kinase (Cell Signaling 2708S), Phospho-S6 Ribosomal Protein (Ser235/236; Cell Signaling 4858S), NRF2 (Cell Signaling 12721), GAPDH (Cell Signaling 2118), HDAC1 (Abcam ab7028). ACE2 antibody (R&D AF933) was used for Flow Cytometry. SARS-CoV-2 Spike antibody (Active Motif, clone AM001414) was used for neutralization at 10 nM. 

### 4.7. ShRNA

For NRF2 knock-down in Calu-3 and Caco2 cells, the sequence of the shRNA targeting 3UTR of the human NRF2 gene was obtained from Sigma Aldrich (St. Louis, MO, USA) (TRCN0000007555; target sequence: GCTCCTACTGTGATGTGAAAT) and cloned in the pTRIP-MND-GFP-H1 lentiviral vector. After lentiviral production and cell transduction, GFP-positive cells were isolated by fluorescence-activated cell sorting and used in functional assays.

### 4.8. Cytokine Measurement

Soluble mediators were detected in Calu-3 culture supernatant by the Proteome Profiler Human XL Cytokine Array Kit (R&D Biosystems, Minneapolis, MN, USA) according to the manufacturer’s instructions. Mouse IFN-beta levels were detected in lung homogenates and in plasma by the ProQuantum Immunoassay Kit (ThermoFisher, Waltham, MA, USA) according to the manufacturer’s instructions.

### 4.9. Primary Cells

Peripheral blood was obtained from healthy donors in accordance with the ethical guidelines at CEA (Fontenay-aux-Roses, France), and Peripheral Blood Mononuclear Cells (PBMC) were isolated by Ficoll (Eurobio Scientific, Les Ulis, France) gradient centrifugation. Monocytes were isolated from PBMC using CD14 microbeads (Miltenyi Biotec, Paris, France) according to the manufacturer’s instructions. PBMC and CD14+ monocytes were cultured in RPMI supplemented with 10% FCS.

### 4.10. Hamsters and In Vivo Infection

Animal housing and experimental procedures were conducted by Oncodesign, according to the French and European Regulations and the Institutional Animal Care and Use Committee of CEA approved by French authorities (CETEA DSV-n°44). The animal BSL3 facility is authorized by the French authorities (Agreement N° D92-032-02). 

6–8 weeks old female Syrian golden hamsters were infected with 10^4^ pfu TCID_50_ of SARS-CoV-2 (Slovakia/SK-BMC5/2020, GISAID EPI_ISL_417879) by intranasal route under a total volume of 70 µL (35 µL per nostril) on Isoflurane-anesthetized animals. Sulfodyne^®^ (600 mg/kg, a dose equivalent to 30 mg/kg of SFN, diluted in 2% ethanol in PBS) or vehicle (2% ethanol in PBS) was administered orally at 8 hpi and then twice daily with an 8 h interval between each delivery on one given day. Body weight was monitored daily. Animals were euthanized at 3 dpi, and the right lung lobe was collected for RNA extraction (Macherey Nagel NucleoSpin) and viral load quantification by qRT-PCR using the IP4 set of primers and probe. 

### 4.11. Mice and In Vivo Infection

B6.129P2-*Nfe2l2^tm1Mym^* (*Nrf2* KO in the text) and C57BL/6J mice were bred at the TAAM (https://www.taam.cnrs.fr/, accessed on 9 July 2024) under SPF conditions. B6.Cg-Tg(K18-ACE2)2Prlmn/J mice (stock #034860, K18-hACE2 in the text) were imported from The Jackson Laboratory (Bar Harbor, ME, USA) and bred at the Institut Pasteur under strict SPF conditions. Infection studies were performed in an animal BSL-3 facility at the Institut Pasteur. All animal work was approved by the Institut Pasteur Ethics Committee (project dap 200008) and authorized by the French Ministry of Research under project 24613 in compliance with European and French regulations.

Infection experiments were performed on male and female mice aged 7–12 weeks (K18-hACE2) or 7–20 weeks (*Nrf2* KO). Groups were composed of sex- and age-matched. Anesthetized (ketamine/xylazine) mice were inoculated intranasally with 6 × 10^4^ plaque-forming units (PFU) of SARS-CoV-2 Beta variant (B.1.351 strain, GISAID ID: EPI_ISL_964916) in a 24 µL volume (*Nrf2* KO) or 10^4^ PFU of SARS-CoV-2 Delta variant (B.1.617.2 strain, GISAID ID: EPI_ISL_3030060) in a 40 µL volume (K18-hACE2). For K18-hACE2 mice experiments, Sulfodyne^®^ (600 mg/kg, a dose equivalent to 30 mg/kg of SFN, diluted in 2% ethanol in PBS) or vehicle (2% ethanol in PBS)) was administered by intraperitoneal injection two times per day, starting one day before infection and for up to 14 days after infection. One group of mice was followed for 14 days post-infection (dpi) for clinical signs and body weight. Each of the four criteria (ruffled fur, hunched posture, reduced locomotion, and difficult breathing) received a 0–2 score and was added to a global score. Mice were euthanized when reaching 25% body weight loss or a global score of 8. Other groups of mice were euthanized at specific dpi for tissue collection.

### 4.12. Viral Load 

Groups of mice were euthanized at 3 or 6 dpi for measurement of viral load and viral titer in lung homogenates and nasal turbinates homogenates. The right lung lobe and nasal turbinates were dissected and frozen at −80 °C. Samples were homogenized in 400 μL of cold PBS using lysing matrix M (MP Biomedical, Irvine, CA, USA) and a MP Biomedical FastPrep 24 Tissue Homogenizer. Viral RNA was extracted in PBS using an extraction robot IDEAL-32 (IDsolutions) and the NucleoMag Pathogen extraction kit (Macherey Nagel). Viral RNA quantification was performed by RT-qPCR using the IP4 set of primers and probe and the Luna Universal Probe One-Step RT-qPCR Kit (NEB). Serial dilutions of a titrated viral stock were analyzed simultaneously to express viral loads as eqPFU per gram of tissue. 

### 4.13. Histopathological Analysis

The left lung lobe was fixed in 10% phosphate-buffered formalin for 7 days. Histological analysis was performed on paraffin-embedded sections and stained with hematoxylin-eosin. The alveolar scoring system considered alveolar thickness, interstitial inflammation, alveolar inflammation, and alveolar inflammation at the surface. The bronchi scoring system considered inflammation and activation. 

### 4.14. Statistical Analysis

Data were analyzed using GraphPad Prism 9. Specific statistical tests are detailed in the figure legends. Statistical significance: * *p* ≤ 0.05; ** *p* ≤ 0.01; *** *p* ≤ 0.001; **** *p* ≤ 0.0001.

## Data Availability

The RNAseq data are available in the GEO database under accession code GSE243268.

## Supplementary Materials

The following supporting information can be downloaded at: https://www.mdpi.com/article/10.3390/antiox13091083/s1.
