# Supplementary material for: Multiple Mechanisms of Action of Sulfodyne®, a Natural Antioxidant, against Pathogenic Effects of SARS-CoV-2 Infection
Source: Antioxidants (Basel). 2024 Sep 4;13(9):1083. doi: 10.3390/antiox13091083 (PMC11429452; doi:10.3390/antiox13091083)
Supplement: Supplementary file 1 [file antioxidants-13-01083-s001.zip › antioxidants-3124985-supplementary.pdf]

Supporting Figure Legends

Figure S1

A) Schematic of experimental design. WT and *Nrf2* KO mice were intranasally infected with the SARS-CoV-2 Beta variant ($6x10^4$ PFU).

B) Viral RNA quantification in homogenized lung tissue at 6 dpi in WT and *Nrf2* KO mice. Individual values for each mouse and median are presented n=13. Two-tailed Mann-Whitney U test.

C) Body weight change during the course of infection presented as a percent change compared to weight measured just before inoculation with SARS-CoV-2. Mean ± sem, n=17 to 30. Mixed-effects model test comparing *Nrf2* KO vs. WT.

D) The clinical score was assessed for ruffled fur, hunched posture, reduced locomotion, and difficulty breathing, with a score ranging from 0 to 2. The cumulative clinical score is indicated. WT mice did not exhibit any clinical signs. Mean ± sem, n=13.

E) Representative haematoxylin-eosin staining (left) and histopathological score distribution (right) of the lungs of WT and *Nrf2* KO mice at 6 dpi. n=12 (WT) or 13 (Nrf2 KO).

* p ≤ 0.05; *** p ≤ 0.001

Figure S2

A) Representative WES Simple assay for NRF2, HDAC1, and GAPDH in nuclear and cytoplasmic extracts from Calu-3 cells treated with Sulfodyne® (50 µg/ml) for 6 h.

B) Cell viability of Calu-3 cells treated with Sulfodyne® for 6 and 30 h, measured by Neutral red assay. Data are presented individually as percent of untreated cells (UT). Mean ± sem, n=6.

C) NRF2-target gene *HMOX1* mRNA levels in Calu-3 infected and treated with Sulfodyne® at indicated times before (-1 h) at the time (0 h) or after infection (+12 h). Dare is shown as fold change over untreated (UT) cells at 36 hpi. Mean ± sem, n=3.

Figure S3

Validation of anti-Spike neutralizing antibody (Spike Ab). SARS-CoV-2 was pre-incubated in the presence or absence of Spike Ab for 1 hour at 37°C before infection of Calu-3 cells. Genomic and sub-genomic viral RNA was measured at 36 hpi. Mean ± sem, n=2.

Figure S4

A) Heatmap of normalized expression levels of DEG (up and down-regulated) in SARS-CoV-2 Calu-3 at 36 hpi vs. mock.

B) Metascape pathway analysis comparing DEG during infection (COV2_36 hpi vs. mock) and DEG by Sulfodyne® treatment (COV2_Sulfodyne®_36 hpi vs. COV2_36 hpi). Circle size indicates the statistical significance of the enrichment.

C-D) Representative WES Simple assay for NRF2 and HDAC1 in a nuclear extract from Calu-3 (C) or Caco2 (D) transduced with shRNA targeting NRF2 (shNRF2) or a non-targeting control (shCT) untreated (UT) or treated with Sulfodyne® for 6 h.

E) Viral genomic and NRF2-target gene *TXNRD1* mRNA levels in shCT and shNRF2 Caco2 infected with SARS-CoV-2 for 48 hpi. Cells were treated with Sulfodyne® at 18 hpi. Data are presented as fold change over ShCT UT cells at 48 hpi for genomic RNA and as fold change over uninfected cells (0 hpi) for *TXNRD1.* Mean ± sem, n=3. Two-way ANOVA with Sidak's multiple comparisons test vs. shCT.

F) WES Simple assay for mTOR substrate proteins and GAPDH in Caco2 cells infected for 48 h and treated with Sulfodyne® or Rapamycin at 18 hpi.

Figure S5

A) Normalized read counts of DPP4 mRNA in Calu-3 cells of indicated RNA-seq samples. n=4.

B) DPP4 mRNA expression (nTPM, normalized transcripts per million) in immune cells showing no expression of DPP4 in monocytes. Underlined in red are $CD14^{+}CD16^{-}$ (classical) monocytes. Datasets were from The Human Protein Atlas database (https://www.proteinatlas.org/).

Figure S6

A) Schematic of experimental design. Syrian Golden hamsters (females, 6-8 weeks old) were intranasally infected with SARS-CoV-2 (10^4 PFU) 8 h before oral administration of Sulfodyne® (600 mg/kg) or vehicle.

B) Lung viral load at 3 dpi in the vehicle and Sulfodyne®-treated hamsters. Mean ± sem, n=6. Two-tailed Mann-Whitney U test.

C) Percent of starting body weight in vehicle and Sulfodyne®-treated hamsters. Mean ± sem, n=6. Two-way ANOVA with Sidak's multiple comparisons tests comparing Sulfodyne® vs. vehicle. * $p \leq 0.05$
