# Supplementary material for: Multiple Mechanisms of Action of Sulfodyne®, a Natural Antioxidant, against Pathogenic Effects of SARS-CoV-2 Infection
Source: Antioxidants (Basel). 2024 Sep 4;13(9):1083. doi: 10.3390/antiox13091083 (PMC11429452; doi:10.3390/antiox13091083)
Supplement: Supplementary file 1 [file antioxidants-13-01083-s001.zip › antioxidants-3124985-supplementary.pptx]

## Slide 1
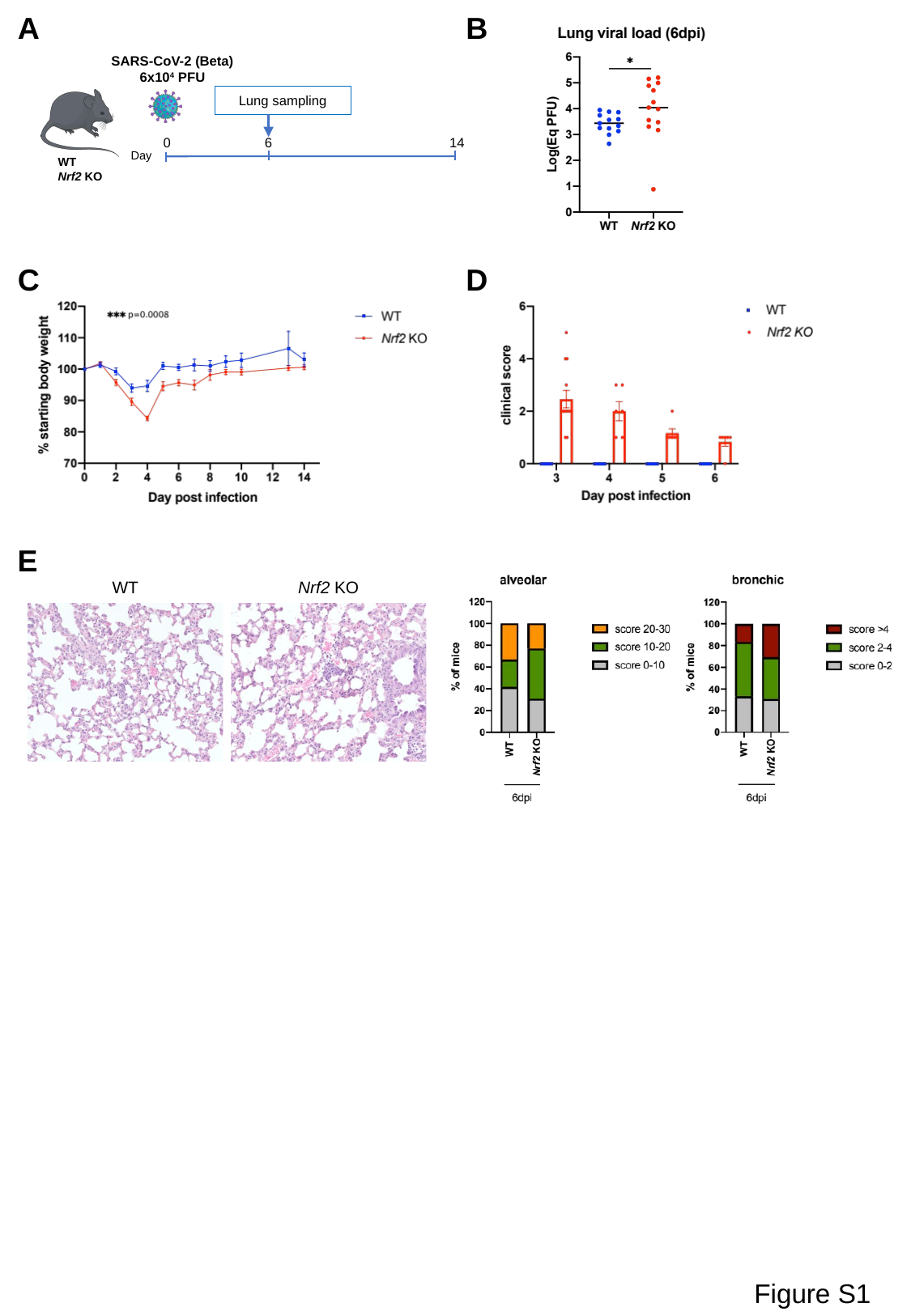

A
B
SARS-CoV-2 (Beta)
6x10^4^ PFU
0
Lung sampling
14
6
Day
WT
Nrf2 KO
C
D
E
WT
Nrf2 KO
Figure S1

## Slide 2
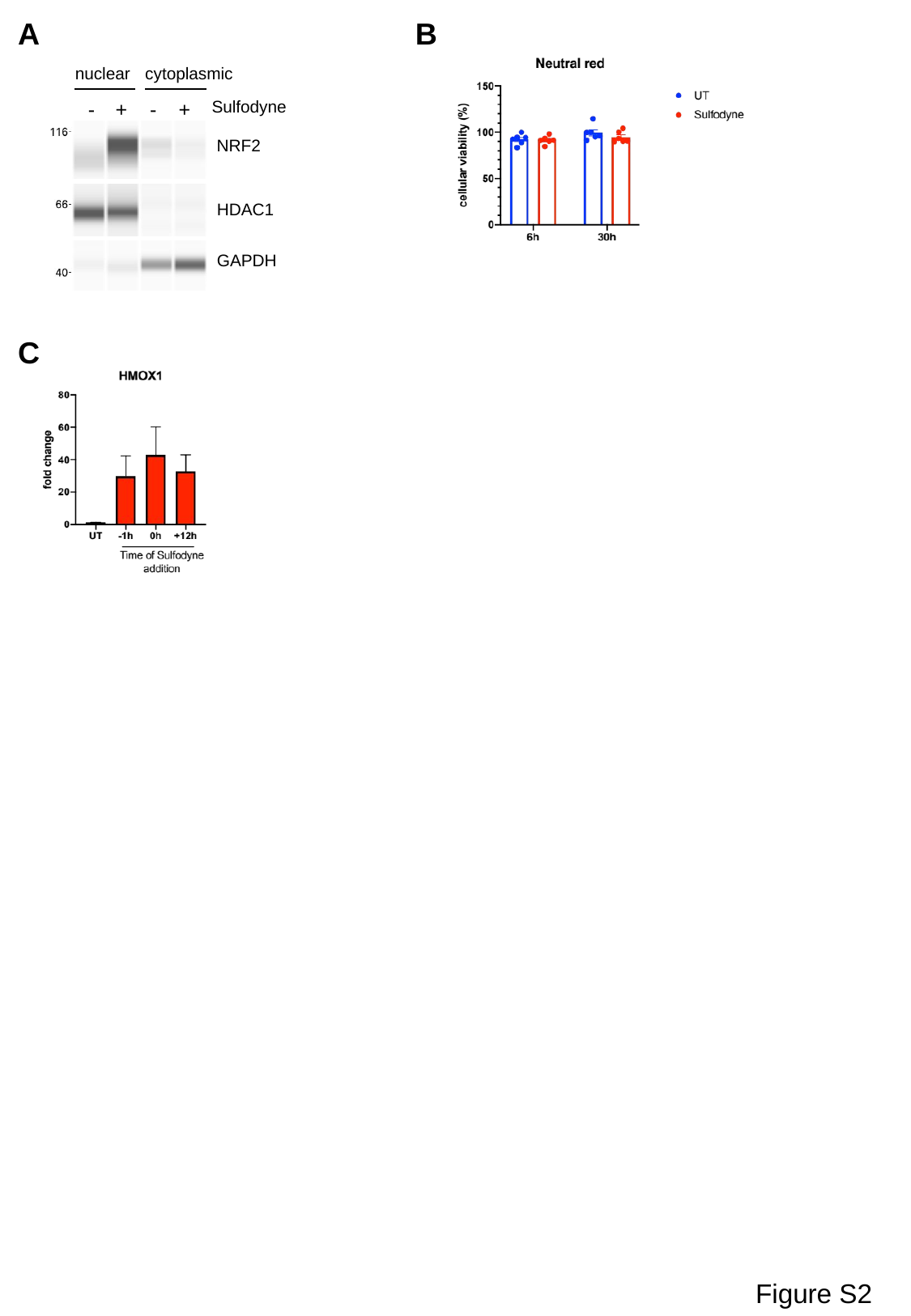

A
B
cytoplasmic
nuclear
Sulfodyne
-
+
-
+
NRF2
HDAC1
GAPDH
C
Figure S2

## Slide 3
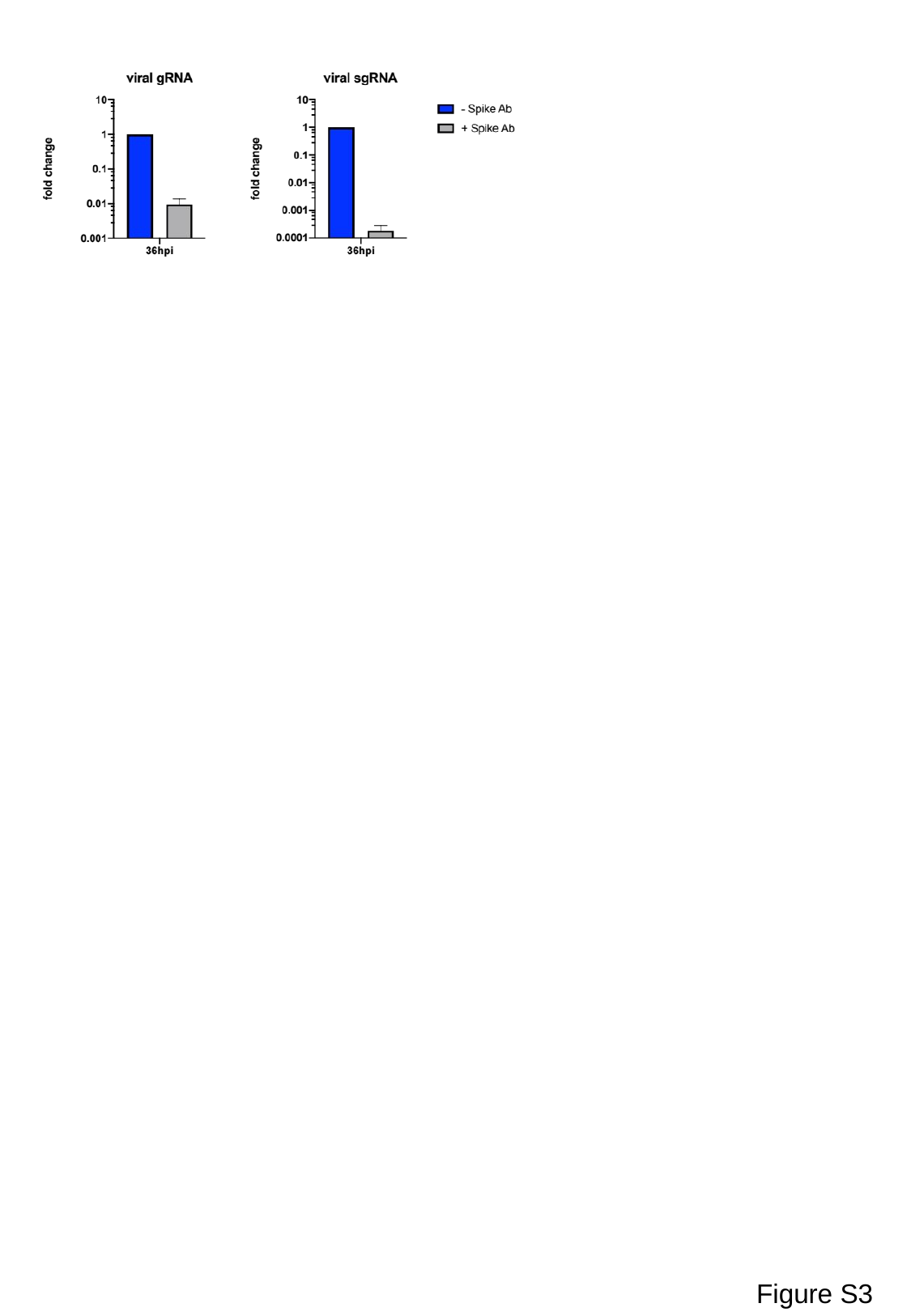

Figure S3

## Slide 4
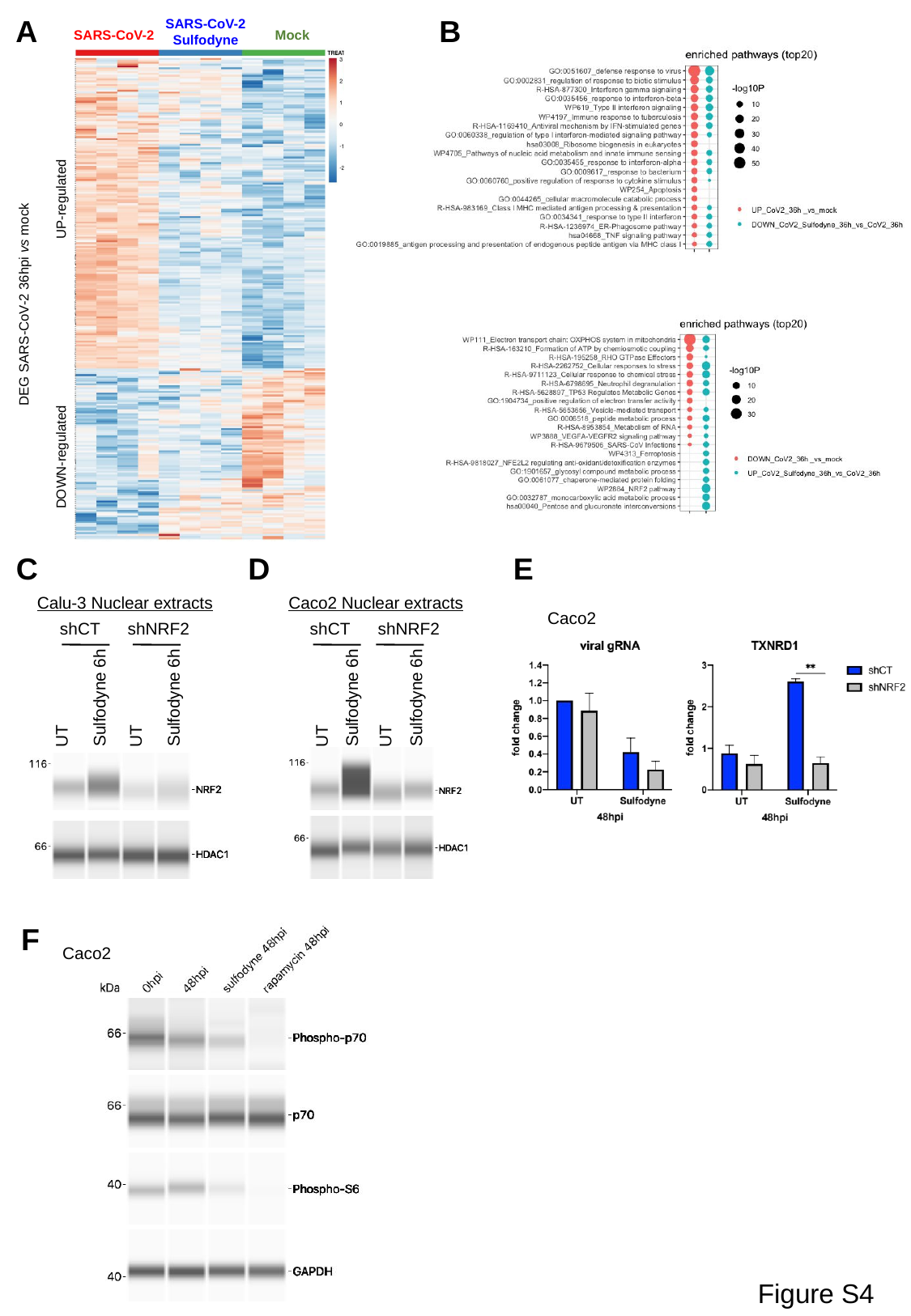

A
B
SARS-CoV-2
Sulfodyne
SARS-CoV-2
Mock
UP-regulated
DOWN-regulated
DEG SARS-CoV-2 36hpi vs mock
C
D
E
Calu-3 Nuclear extracts
Caco2 Nuclear extracts
Caco2
shCT
shNRF2
shCT
shNRF2
Sulfodyne 6h
UT
Sulfodyne 6h
UT
Sulfodyne 6h
UT
Sulfodyne 6h
UT
F
Caco2
Figure S4

## Slide 5
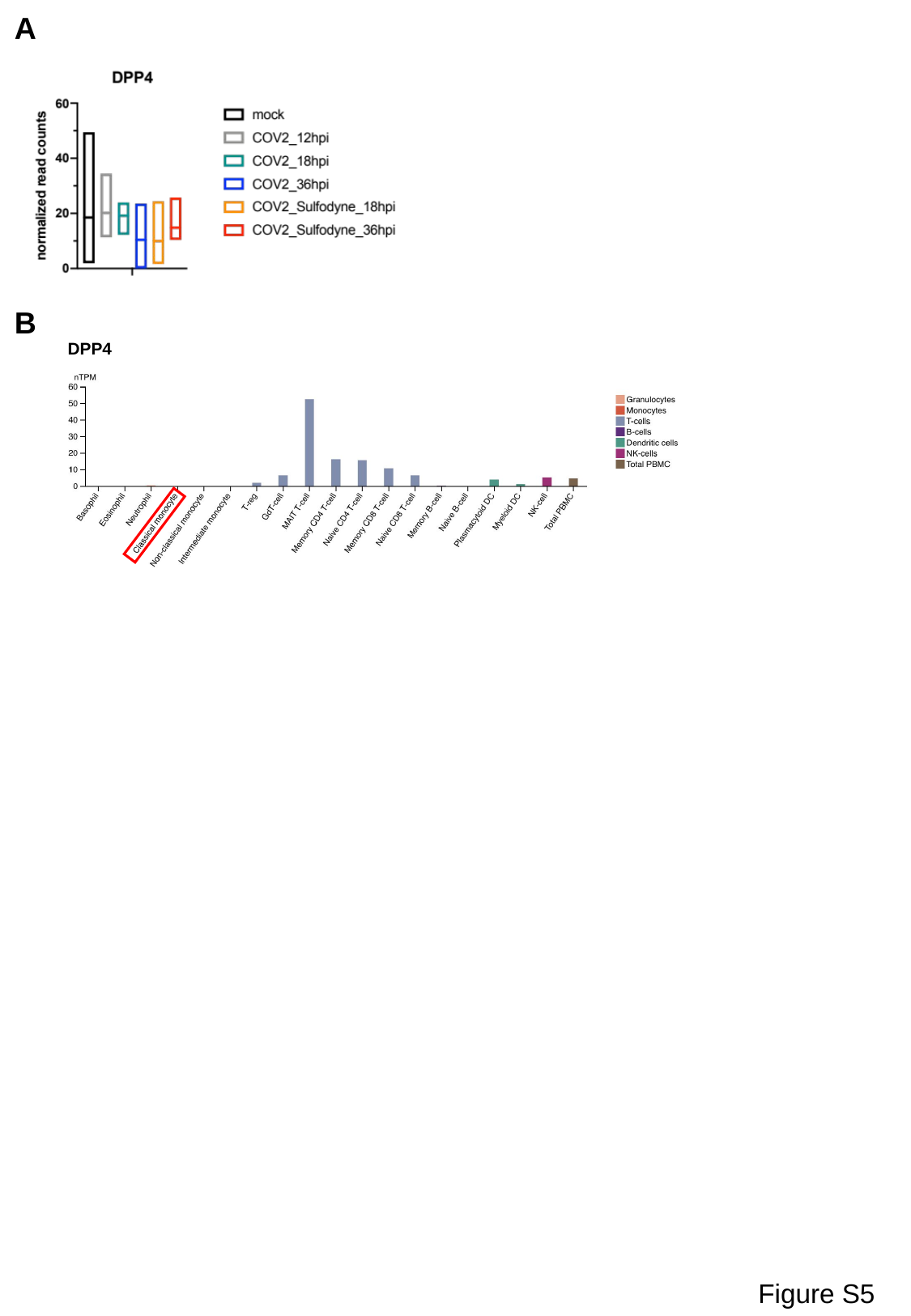

A
B
DPP4
Figure S5

## Slide 6
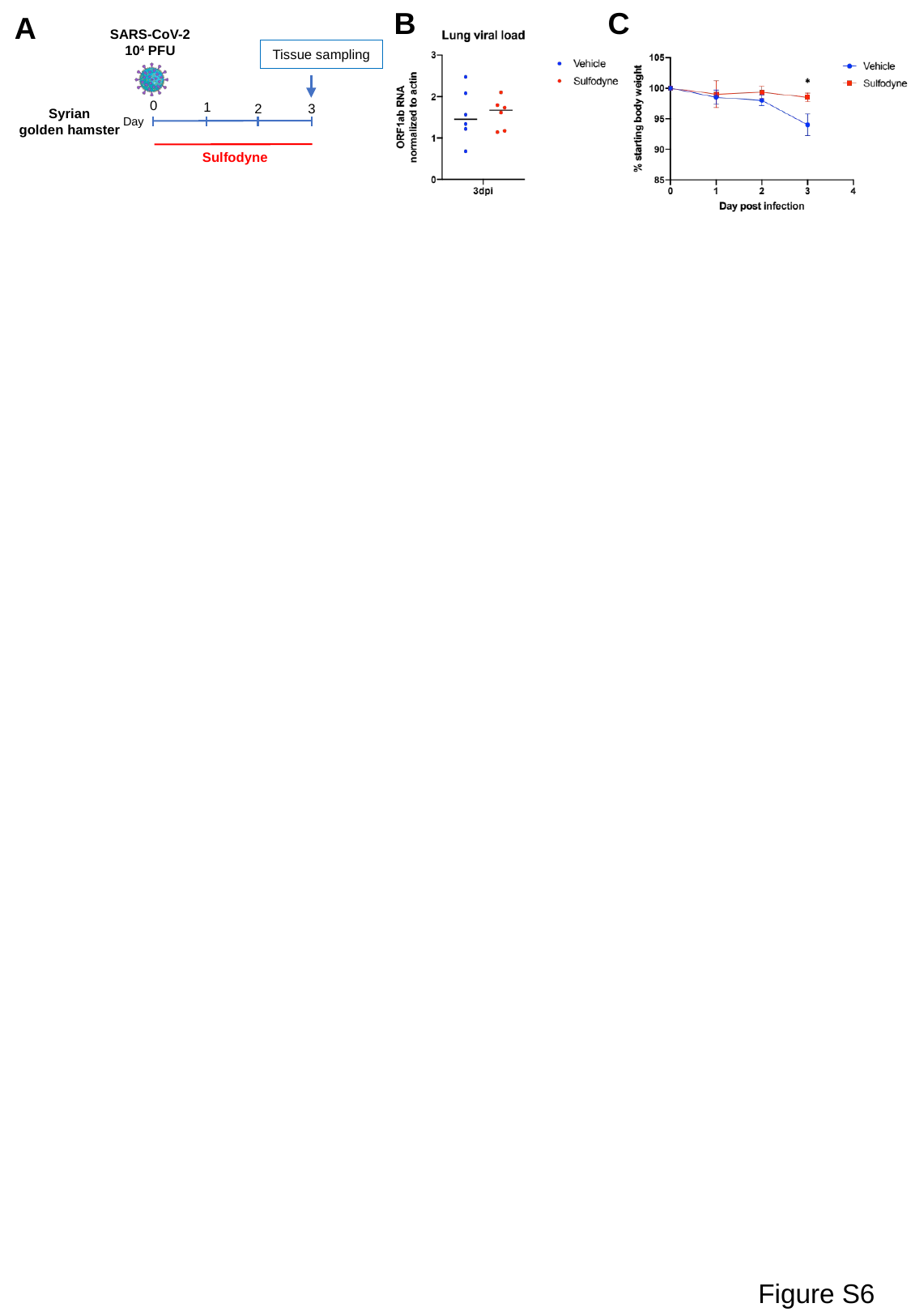

B
C
A
SARS-CoV-2
104 PFU
0
1
3
Tissue sampling
2
Syrian
golden hamster
Day
Sulfodyne
Figure S6
